# *CircGLIS3* Inhibits Intramuscular Adipogenesis and Alleviates Skeletal Muscle Fat Infiltration

**DOI:** 10.1002/jcsm.70009

**Published:** 2025-07-30

**Authors:** Shengchen Yu, Jianfang Wang, Haibing Liu, Juntao Guo, Abebe Belete Kuraz, Yueting Pan, Anning Li, Chugang Mei, Gong Cheng, Linsen Zan

**Affiliations:** ^1^ College of Animal Science and Technology Northwest A&F University Yangling China; ^2^ College of Grassland Agriculture Northwest A&F University Yangling China; ^3^ National Beef Cattle Improvement Center Yangling China

**Keywords:** AMPK signalling pathway, *circGLIS3*, fat infiltration, intramuscular adipogenesis

## Abstract

**Background:**

Intramuscular fat (IMF) is a key determinant of meat quality. Excessive IMF deposition, commonly observed in human obesity and aging, negatively affects skeletal muscle function. Circular RNAs (circRNAs) play critical regulatory roles in muscle and fat development, as well as in the progression of related diseases. However, the specific functions and underlying mechanisms of circRNAs in IMF deposition have not been extensively studied.

**Methods:**

We screened adipogenic differentiation‐related circRNAs using circRNA‐seq combined with WGCNA. Functional analyses, including loss‐of‐function and gain‐of‐function experiments, were performed to determine the role of *circGLIS3* in regulating adipogenesis in bovine intramuscular preadipocytes and 3T3‐L1 cells. A high‐fat diet‐induced mouse model was established to investigate the in vivo effects of *circGLIS3* on skeletal muscle fat infiltration in mice (C57BL/6; *n* = 6 males). Mechanistic studies involved transcriptomic analysis, dual‐luciferase reporter assays, RNA immunoprecipitation, and rescue experiments.

**Results:**

We screened and characterized a novel circRNA, *circGLIS3*, which is highly expressed in bovine IMF tissue and in the skeletal muscle of mice exhibiting fat infiltration. Functionally, *circGLIS3* overexpression inhibited triglyceride synthesis (−16.19%, *p* < 0.01) and lipid droplet accumulation (−42.44%, *p* < 0.01) in intramuscular preadipocytes, concurrently downregulating the expression of key adipogenesis‐related genes and proteins (*p* < 0.05). *CircGLIS3* knockdown promoted triglyceride synthesis (+28.32%, *p* < 0.05) and lipid droplet accumulation (+47.40%, *p* < 0.01) in intramuscular preadipocytes, accompanied by a significant upregulation of adipogenesis‐related genes and proteins (*p* < 0.05). Notably, *circGLIS3* exhibits more than 80% sequence homology among bovine, mouse, and human species. *CircGLIS3* overexpression inhibited triglyceride synthesis (−28.84%, *p* < 0.01) and lipid droplet accumulation (−46.49%, *p* < 0.01) in 3T3‐L1 cells, concurrently downregulating the expression of adipogenesis‐related genes and proteins (*p* < 0.05). Overexpression of *circGLIS3* reduced skeletal muscle fat infiltration induced by high‐fat diet in mice (−57.88%, *p* < 0.05). Mechanistically, *circGLIS3* acts as a sponge for miR‐21‐3p, increasing the expression of its target gene, *LEPR* (+56.05%, *p* < 0.01), and promoting the phosphorylation level of its downstream signalling protein, AMPKα (+68.68%, *p* < 0.01).

**Conclusions:**

*CircGLIS3* inhibits bovine intramuscular adipogenesis by regulating the miR‐21‐3p/LEPR/AMPK axis, reducing fat infiltration in mouse skeletal muscle. These findings suggest that *circGLIS3* is a promising target for improving meat quality in livestock, and as a potential therapeutic marker for alleviating skeletal muscle fat infiltration associated with obesity.

## 1 Introduction

Skeletal muscle fat infiltration, frequently associated with the accumulation of intramuscular fat (IMF) and intramuscular connective tissue, is a common complication of human obesity that can lead to sarcopenia and muscular dystrophy [1]. It is well known that excessive fat accumulation in skeletal muscle can weaken muscle strength [2], disrupt insulin sensitivity [3], and lipid metabolism [4], and even exert feedback effects on systemic metabolism [5]. IMF content is a key factor in meat quality [6], positively influencing tenderness, juiciness, flavour and nutrition, but excessive IMF can increase toughness, reducing overall meat quality [7]. Research indicates that IMF is a highly heritable trait among different species [6], which implies that understanding its genetic mechanisms could improve meat quality and address fat infiltration in human skeletal muscle through molecular regulatory pathways.

CircRNAs, a unique class of endogenous ncRNAs present in eukaryotic cells with a closed‐loop structure, are conserved across species and exhibit tissue‐ or stage‐specific expression [8]. Their unique structure gives them a longer half‐life and stronger adaptability to achieve unique biological functions [9]. There is increasing evidence that circRNAs can exert their biological functions by acting as sponges for miRNAs and RNA‐binding proteins, regulating transcription and splicing, and serving as templates for translation. These biological functions play significant roles in fat and muscle development [10, 11], disease treatment [12] and cancer progression [13]. In skeletal muscle, *circCPE* [14], *circNEB* [15] and *circZNF609* [11] are involved in the regulation of myogenesis. In addition, *circHIPK3* facilitates the repair of ischaemic muscle injuries [16], while *circTmeff1* promotes muscle atrophy [17]. While extensive research has established the significant regulatory roles of circRNAs in muscle development, disease and therapeutic strategies, studies exploring their impact on improving meat quality or muscle‐related diseases via the regulation of IMF deposition remain scarce. Therefore, it is necessary to explore the regulatory mechanisms of circRNAs in intramuscular adipogenesis.

IMF originates from fibro/adipogenic progenitors (FAPs), a type of bipotent progenitor cell capable of differentiating into preadipocytes and myofibroblasts, which are the primary cells types for adipose and fibrotic tissue formation, respectively. Wang et al. [18] performed single‐cell sequencing of skeletal muscle from three beef cattle breeds and analysed it in integration with multiple published datasets from humans, monkeys and mice. The results indicate a greater similarity in FAPs among large mammals. In this study, we screened a novel circRNA, *circGLIS3*, through circRNA sequencing (circRNA‐seq) data analysis combined with weighted gene co‐expression network analysis (WGCNA). *CircGLIS3* acts as a competitive endogenous RNA (ceRNA) to regulate leptin receptor (*LEPR*) expression by competitively sponging miR‐21‐3p, which activates the adenosine 5′‐monophosphate‐activated protein kinase (AMPK) signalling pathway and ultimately inhibits intramuscular adipogenesis. These study aims to elucidate the role and mechanism of circRNAs in bovine intramuscular adipogenesis, providing new insights for enhancing beef meat quality, directional selection in beef cattle, and molecular breeding strategies. In vivo experiments in mice further verified that *circGLIS3* can inhibit high‐fat diet (HFD)‐induced IMF deposition in skeletal muscle, suggesting its potential as a therapeutic target for addressing obesity‐related inflammation in human skeletal muscle.

## 2 Materials and Methods

### 2.1 Ethics Statement

All experimental animal protocols were approved by the Committee of Experimental Animal Management at Northwest A&F University, China (Approval ID: DK2021042) and complied with applicable regulations.

### 2.2 WGCNA Analysis

Our previous research performed circRNA‐seq at four stages of adipogenic (0, 3, 6 and 9 days after differentiation) from bovine intramuscular preadipocytes [19]. WGCNA analysis was performed as described in previous studies [20], with detailed information provided in the Supporting Information.

### 2.3 Tissue Sample Preparation

Different tissue samples were collected from three adult Qinchuan beef cattle (2‐year‐old). IMF tissues were stripped from the skeletal muscle of beef cattle at different ages (1, 2, 3 years old, *n* = 3). Samples were rapidly frozen in liquid nitrogen and stored at ‐80 °C.

### 2.4 Cell Isolation, Culture and Differentiation

Primary bovine intramuscular preadipocytes were isolated from the skeletal muscle of three newborn cattle, as previously described [21]. Primary bovine intramuscular preadipocytes and 3T3‐L1 cells (ATCC CL‐173, established mouse preadipocyte cell line) were used to study the functions and mechanisms of adipogenesis. HEK293T cells (ATCC CRL‐11268, established human embryonic kidney 293 cell line) were used for the dual‐luciferase reporter assay. The detailed information is provided in the Supporting Information.

### 2.5 Cell Transfection

The full‐length oligonucleotide sequence of *circGLIS3* was synthesized and cloned into the pcD25‐ciR and pcD5‐ciR vectors (Geneseed, China). The small interfering RNA (siRNA) was synthesized by GenePharma Biol. Details of the other transfection materials are described in the Supporting Information. Lipofectamine 3000 reagent (Invitrogen, United States) was used for all transient transfections according to the manufacturer's instructions.

### 2.6 RNA Preparation and qRT‐PCR Analysis

According to the manufacturer's instructions, total RNA was extracted using RNAiso Plus (Takara, China). Total RNA was reverse‐transcribed with the Prime Script TMRT Reagent Kit with gDNA Eraser (Takara) or the miRcute Plus miRNA First‐Strand cDNA Kit (TianGen, China). The qRT‐PCR analysis was performed using SYBR Premix ExTaq II (TaKaRa) or the miRcute miRNA qRT‐PCR Detection Kit (TianGen) on the CFX Connect System (Bio‐Rad, United States) [22]. The expression levels of circRNAs, mRNAs and miRNAs were quantified using the 2^−ΔΔCT^ method with β‐actin or U6 as the internal control. The qRT‐PCR primers are listed in Tables S3 and SS4.

### 2.7 Oil Red O Staining and Determination of Triglyceride Content

The mature adipocytes were fixed in 4% paraformaldehyde for 20 min and subsequently stained with Oil Red O for 30 min. Subsequently, the cells were imaged using an optical microscope (Olympus, Japan) and then processed and analysed via ImageJ software (National Institutes of Health, United States). The triglyceride content was quantified using a triglyceride enzymatic assay kit (Applygen, China) according to the manufacturer's instructions.

### 2.8 Western Blot (WB) Analysis

WB analysis was performed as previously described [21]. The images were captured via a Bio‐Rad Molecular Imager (Bio‐Rad, United States) and then processed and analysed via ImageJ software. Antibody information is provided in Table S6.

### 2.9 In Vivo Animal Studies

C57BL/6 mice (8‐weeks old, approximately 20 g) were purchased from SPF Biotechnology Co. Ltd. First, a HFD‐induced mouse skeletal muscle fat infiltration model was established. Following this, pcD5‐circGLIS3 or pcD5‐NC was injected into the vastus lateralis (VL) every 5 days for a total of 30 days. At each injection time point, each mouse was weighed individually, and both the provided feed and the remaining feed were weighed to determine daily feed intake. After the injection experiment, the VL muscles were harvested for histology analysis [23], qRT‐PCR and WB analysis. The detailed information is provided in the Supporting Information.

### 2.10 RNA Sequencing (RNA‐Seq) Analysis

Total RNA was sequenced using the Illumina NovaSeq 6000 platform (Lc‐Bio, China). Differentially expressed genes (DEGs) were identified using DESeq2 (|fold change| > 2, FDR < 0.05). KEGG enrichment analysis was performed using OmicStudio tool. The detailed information is provided in the Supporting Information.

### 2.11 Bioinformatic Target Prediction

The miRNA binding capacity of *circGLIS3* was analysed using prediction tools such as the miRDB (http://mirdb.org/custom.html) and BiBiServ (https://bibiserv.cebitec.uni‐bielefeld.de/rnahybrid) websites. miR‐21‐3p targets were predicted using miRWalk (http://mirwalk.umm.uni‐heidelberg.de/) and TargetScan 8.0 (http://www.targetscan.org/).

### 2.12 Other Molecular Experiments

The procedures used for nuclear‐cytoplasmic separation, RNA‐FISH, histology, dual‐luciferase reporter and RIP assay are described in the Supporting Information.

### 2.13 Statistical Analysis

All the data were provided as the mean ± standard deviation (SD) and analysed using the Student's *t* test, one‐way ANOVA analysis and Duncan's multiple comparison test in IBM SPSS Statistics 26.0. The construction of images was performed using GraphPad Prism 8.0.2. The significance levels **p < 0.01 or *p < 0.05 defined the differences as either very significant or significant.

## 3 Results

### 3.1 WGCNA Analysis and Screening of Candidate circRNAs

**FIGURE 1:**
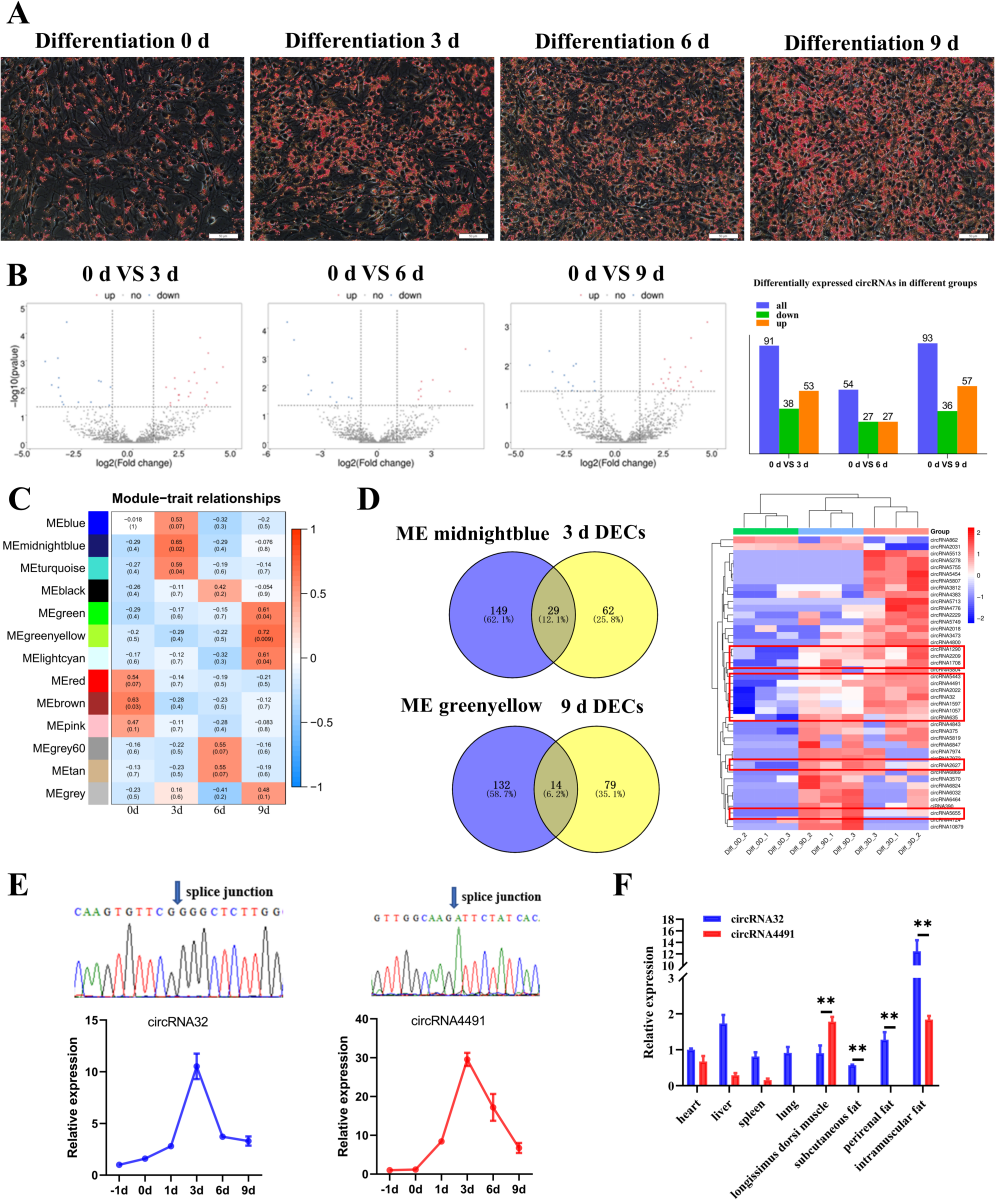
Screened circRNAs are significantly associated with intramuscular preadipocyte deposition. (A) Oil Red O (scale bar 50 μm) staining of primary bovine intramuscular preadipocytes at four adipogenesis differentiation stages (0, 3, 6 and 9 days). (B) Volcano plots of differentially expressed circRNAs (DECs). Statistical results of DECs. (C) Module‐trait relationship heatmap showing the correlation coefficient between each module and the four adipogenesis stages, including 0, 3, 6, and 9 days after differentiation. The colour gradient represents the strength and direction of pairwise correlations between gene modules or samples: Red indicates a strong positive correlation, with a correlation coefficient close to +1 for redder colours. Blue indicates a strong negative correlation, with a correlation coefficient close to −1 for bluer colours. White is an intermediate colour, representing weak or near‐zero correlations. (D) Overlapping analysis of circRNAs between DECs (3 days) and the co‐expression midnightblue module (above), and circRNAs between DECs (9 days) and co‐expression greenyellow module (below), and created a heatmaps of these circRNAs. (E) Sanger sequencing of candidate circRNAs confirmed the back‐splicing junction sequence (above), and qRT‐PCR (*n* = 6) detected the expression of candidate circRNAs in different developmental stages of primary bovine intramuscular preadipocytes (below). (F) The expression of *circRNA32* and *circRNA4491* was detected by qRT‐PCR (*n* = 3) in different tissues of the adult cattle (2‐year‐old). Results are presented as the means ± SD; *p < 0.05, **p < 0.01.

The number of lipid droplets in primary bovine intramuscular preadipocytes progressively increased with the progression of adipogenic differentiation (Figure 1A). To reveal the role of circRNAs in intramuscular adipogenic differentiation, our laboratory performed circRNA‐seq analysis on primary bovine intramuscular preadipocytes at four stages of adipogenic differentiation (0, 3, 6 and 9 days after differentiation) [19]. The visualization results of the differentially expressed circRNAs (DECs) (Additional file 1) are shown in Figure 1B. The KEGG enrichment results showed that the parental genes of DECs were mainly enriched in pathways related to adipogenic differentiation, including PPAR, adipocytokine and MAPK signalling pathway (Figure S1A, Additional file 2).

To identify circRNAs closely associated with intramuscular adipogenesis, we constructed a gene co‐expression network using WGCNA analysis. The hierarchical clustering results showed no outliers among the samples, and individual samples could be used to construct a WGCNA (Figure S1B). We chose the soft‐threshold 8 (based on the scale‐free topology criterion with *R* ^2^ = 0.85) to obtain a scale‐free network (Figure S1C). The adjacency matrix was converted into a TOM matrix, which was used to show the similarity between nodes by considering the weighted correlation. Next, the dynamic hybrid shear method of module partitioning initially yielded 19 co‐expression modules, which were merged into modules with high ME similarity (modules below the red line, line = 0.5), and finally identified 13 co‐expression modules (Figure S1D, Additional file 3). Genes characterized by modules closely associated with different differentiation stages of intramuscular preadipocytes were calculated by Pearson correlation analysis, and correlation heatmaps were drawn, as shown in Figures S1E and 1C. Based on the heatmap in Figure 1C, we selected modules with a correlation higher than 0.65 as those closely associated with intramuscular preadipocyte differentiation. Among them, the midnightblue module was closely related to adipocytes differentiated for 3 days (diff‐3d), and the greenyellow module was closely related to adipocytes differentiated for 9 days (diff‐9d). Through overlap analysis, we identified 29 DECs in the midnightblue module and 14 DECs in the greenyellow module, generated a heatmap of these circRNAs (Figure 1D). Subsequently, we selected 12 circRNAs that were stably and highly expressed in diff‐3d and diff‐9d as candidate circRNAs.

Sanger sequencing identified 8 circRNAs with back‐splicing sites (Figures 1E and S2A–F). Temporal expression analysis showed that the expression of *circRNA32* and *circRNA4491* displayed the most significant changes on the third day of differentiation, exhibiting approximately 10‐fold and 30‐fold increases, respectively, compared to pre‐differentiation levels (Figure 1E). The expression of *circRNA32* was higher than that of *circRNA4491* in the subcutaneous fat, perirenal fat and IMF of adult cattle (Figure 1F). Therefore, *circRNA32* was selected as a potential candidate circRNA to investigate intramuscular adipogenesis.

### 3.2 Characterization of *cir*cGLIS3 in Primary Bovine Intramuscular Preadipocytes

**FIGURE 2:**
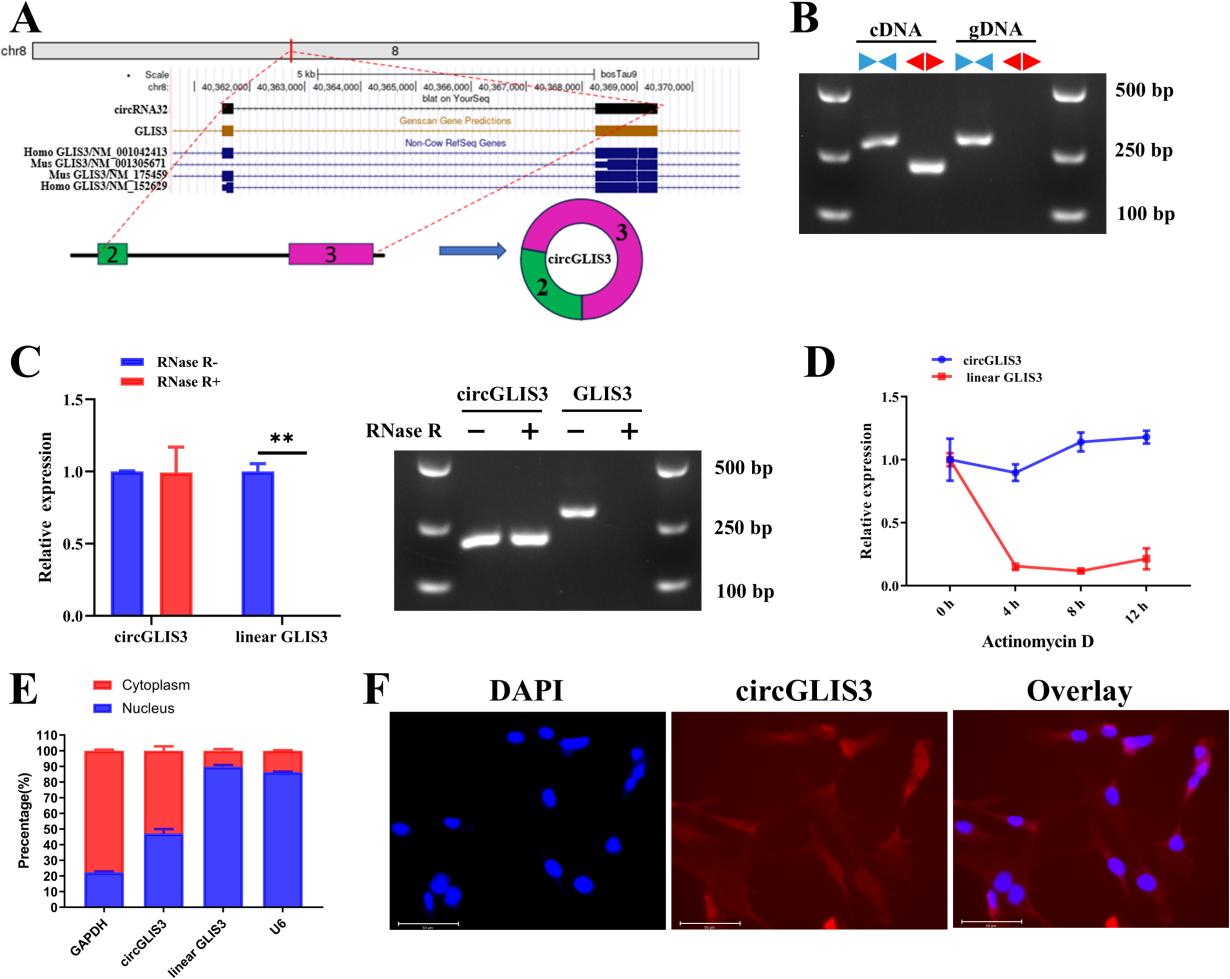
Characterization of *circGLIS3* in primary bovine intramuscular preadipocytes. (A) Schematic diagram of circularization of exons 2 and 3 of the *GLIS3* gene to form *circGLIS3*. (B) Convergent primers and divergent primers were used to confirm the circular nature of *circGLIS3* by agarose gel electrophoresis. (C,D) Resistance of *circGLIS3* and *GLIS3* to RNase R (C) and actinomycin D (D) was tested by qRT‐PCR (*n* = 6) or agarose gel electrophoresis. (E,F) The distribution of *circGLIS3* in the cytoplasm and nuclei of intramuscular preadipocytes was determined by the nucleoplasmic separation experiments (*n* = 6) (E) and RNA fluorescence in situ hybridization assay (RNA‐FISH, scale bar 10 μm) (F). *GAPDH* and *U6* were used as cytoplasmic and nuclear localization controls, respectively. Results are presented as the means ± SD; *p < 0.05, **p < 0.01.

The *circRNA32* (1334 bp in length) was named *circGLIS3*, because it is derived from the second and third exons of the maternal gene GLIS family zinc finger 3 (*GLIS3*), located on chromosome 8 (Figure 2A), a candidate gene for type 2 diabetes. Agarose gel electrophoresis results showed that convergent primers could amplify the target sequence from both cDNA and gDNA templates, whereas divergent primers could only amplify the target sequence from the cDNA template (Figure 2B). The RNase R tolerance test and actinomycin D assay showed that *circGLIS3* exhibited greater stability compared to linear mRNA (Figure 2C,D). Nuclear‐cytoplasmic separation and RNA‐FISH assays results indicated that *circGLIS3* were predominantly located in the cytoplasm (Figure 2E,F).

### 3.3 *CircGLIS3* Inhibits Adipogenesis in Primary Bovine Intramuscular Preadipocytes

**FIGURE 3:**
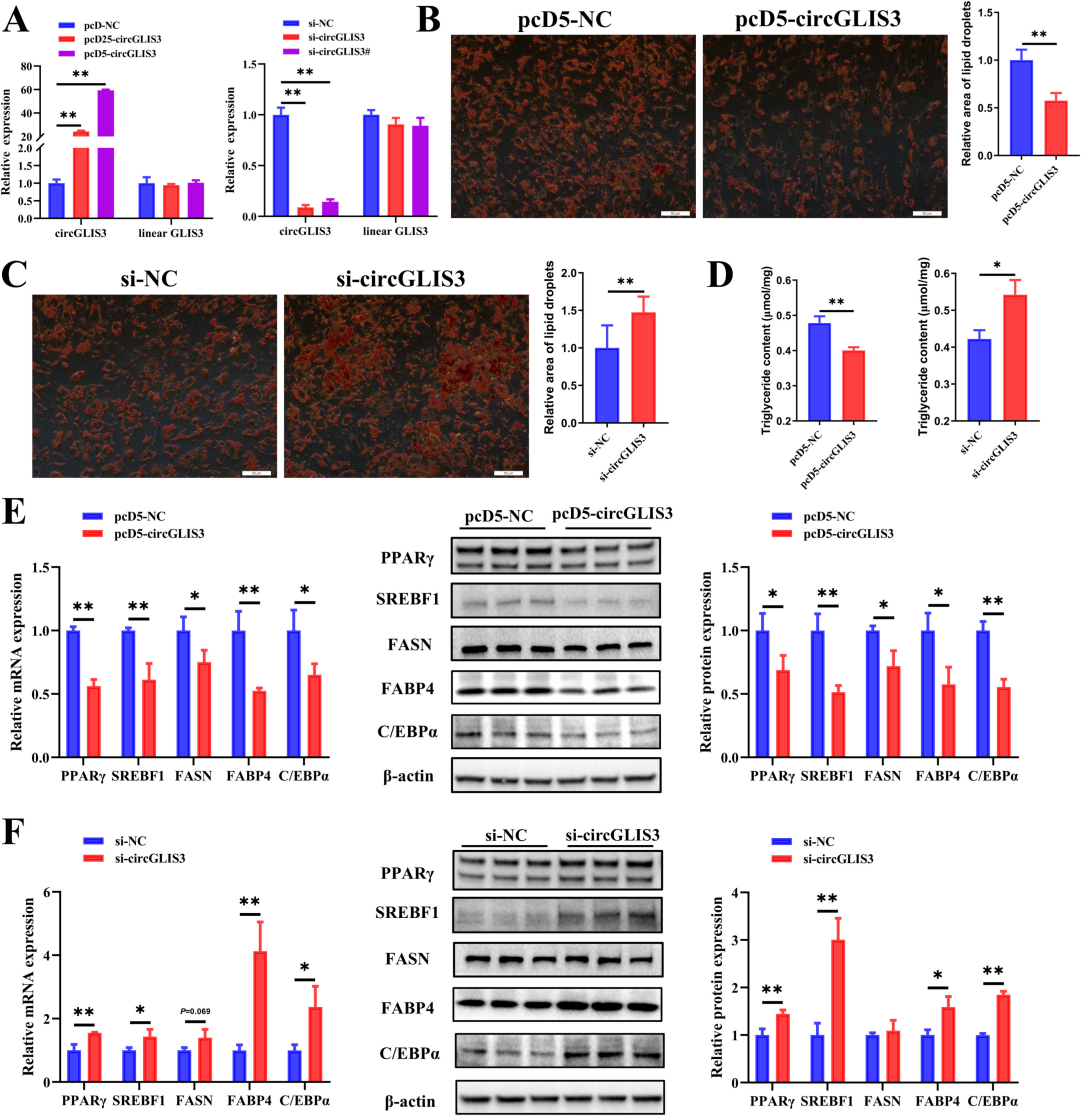
*CircGLIS3* inhibits primary bovine intramuscular adipogenesis. (A) The expression efficiency of *circGLIS3* interference (left) and overexpression (right) was determined by qRT‐PCR (*n* = 6) on the third day of differentiation after transfection with pcD5‐circGLIS3 or si‐circGLIS3. (B,C) Oil Red O staining (*n* = 9, scale bar 50 μm) evaluated the lipid droplet content of intramuscular adipocytes on the sixth day of differentiation. (D) Determination of triglyceride content (*n* = 3) on the sixth day of differentiation. (E,F) Relative mRNA and protein expression levels of PPARγ, SREBF1, FASN, FABP4 and C/EBPα were analysed by qRT‐PCR (*n* = 6) and Western blot (*n* = 3) on the third day of differentiation. Results are presented as the means ± SD; *p < 0.05, **p < 0.01.

To explore the function of *circGLIS3* in intramuscular adipogenesis, we first investigated whether *circGLIS3* affects the adipogenic differentiation of primary bovine intramuscular preadipocytes. Transfection results showed that the overexpression efficiency of pcD5‐circGLIS3 and the interference efficiency of si‐circGLIS3 were relatively high, with no significant effect on linear *GLIS3* mRNA expression (Figure 3A), and were chosen for subsequent analysis. Further experiments demonstrated that *circGLIS3* overexpression significantly reduced the levels of triglycerides and lipid droplets, while knockdown of *circGLIS3* showed the opposite effect (Figure 3B–D). Since *circGLIS3* expression peaked on the third day of intramuscular preadipocyte differentiation (Figure 1E), adipogenesis‐related markers were analysed by qRT‐PCR and WB on the third day of differentiation. *CircGLIS3* overexpression significantly suppressed the mRNA and protein expression of peroxisome proliferator‐activated receptor gamma (PPARγ), sterol regulatory element binding transcription factor 1 (SREBF1), fatty acid synthase (FASN), fatty acid binding protein 4 (FABP4) and CCAAT/enhancer binding protein alpha (C/EBPα) in intramuscular preadipocytes (Figure 3E). In contrast, *circGLIS3* interference yielded the opposite result (Figure 3F). Collectively, these findings demonstrate that *circGLIS3* inhibits intramuscular adipogenesis.

### 3.4 *CircGLIS3* Inhibits Adipogenesis in the 3T3‐L1 Mouse Preadipocyte Cell Line

**FIGURE 4:**
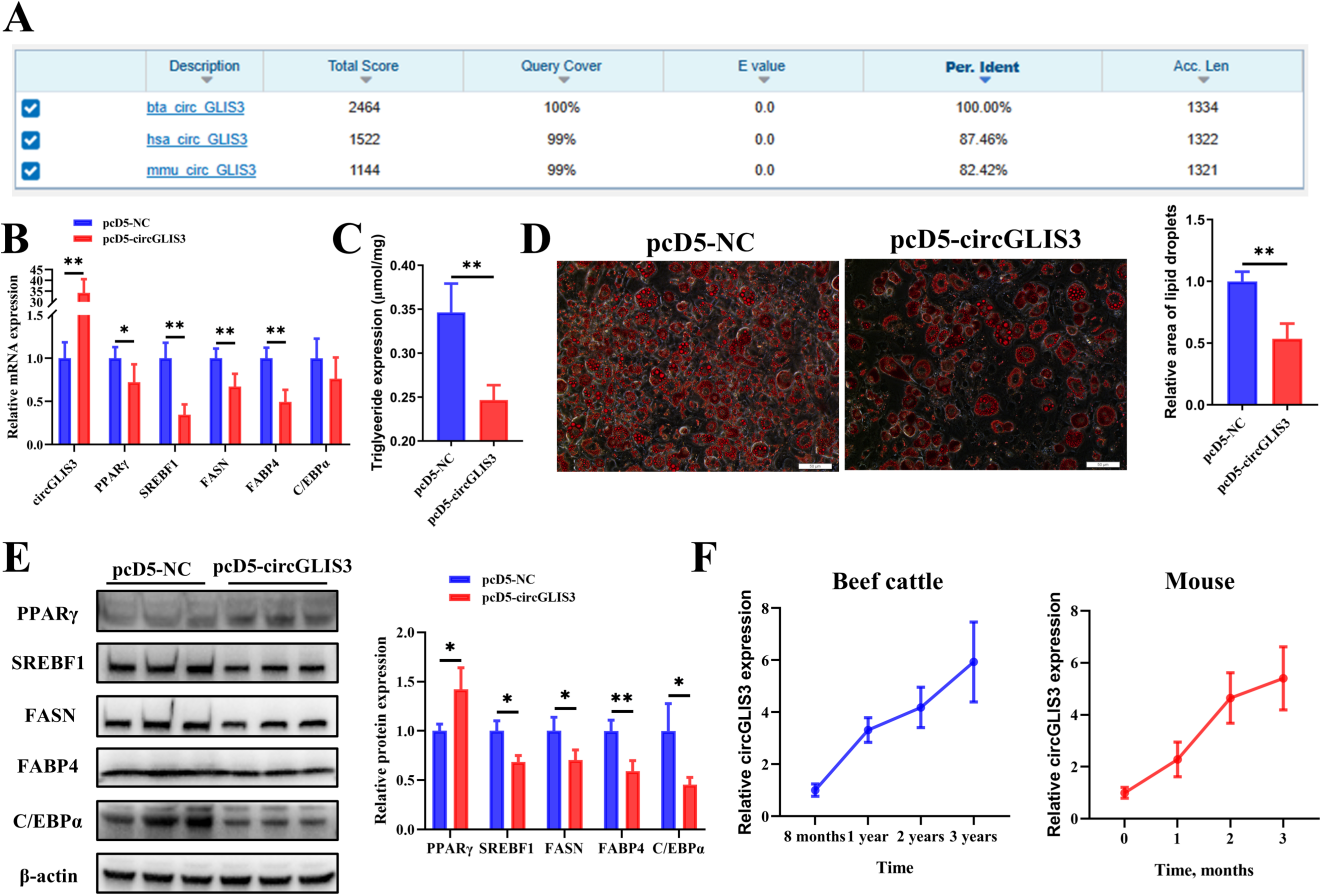
*CircGLIS3* inhibits adipogenesis in the 3T3‐L1 mouse preadipocyte cell line. (A) Analysis of the sequence homology of *circGLIS3* among cattle, mice, and humans using NCBI BLAST. (B) Relative mRNA expression levels of *circGLIS3*, *PPARγ*, *SREBF1*, *FASN*, *FABP4* and *C/EBPα* were analysed by qRT‐PCR (*n* = 6) on the third day of differentiation. (C) Determination of triglyceride content (*n* = 3) on the sixth day of differentiation. (D) Oil Red O staining (*n* = 9, scale bar 50 μm) evaluated the lipid droplet content of intramuscular adipocytes on the sixth day of differentiation. (E) Protein expression levels of PPARγ, SREBF1, FASN, FABP4 and C/EBPα were analysed by Western blot (*n* = 3) on the third day of differentiation. (F) *CircGLIS3* expression in IMF tissue of Qinchuan beef cattle at different ages was detected by qRT‐PCR (*n* = 3). Two‐month‐old mice were fed a high‐fat diet, and the skeletal muscle tissues were collected at 1, 2 and 3 months afterward to measure *circGLIS3* expression by qRT‐PCR (*n* = 6). Results are presented as the means ± SD; *p < 0.05, **p < 0.01.

It is well known that mice are the most widely used model animals for research into biological functions and regulatory mechanisms. We performed a homology search of *circGLIS3* from mice and cattle using NCBI BLAST, and the results showed a similarity of over 80% (Figure 4A, Additional files [Link], [Link], [Link], [Link]). We transfected the bovine *circGLIS3* overexpression plasmid into 3T3‐L1 cells and found that its effect on adipogenesis in 3T3‐L1 cells was consistent with its effect in bovine intramuscular preadipocytes (Figure 4B–E). To determine whether *circGLIS3* plays a role in IMF deposition, we measured *circGLIS3* expression levels in the IMF tissue of beef cattle at different ages (1, 2, 3 years) and in the skeletal muscle of mice at different time points (1, 2 and 3 months on high‐fat feeding) (Figure 4F). Results showed that the expression level of *circGLIS3* gradually increased with the IMF accumulation, indicating that *circGLIS3* plays an important role in the process of IMF deposition.

### 3.5 *CircGLIS3* Alleviates Fat Infiltration in Mouse Skeletal Muscle

**FIGURE 5:**
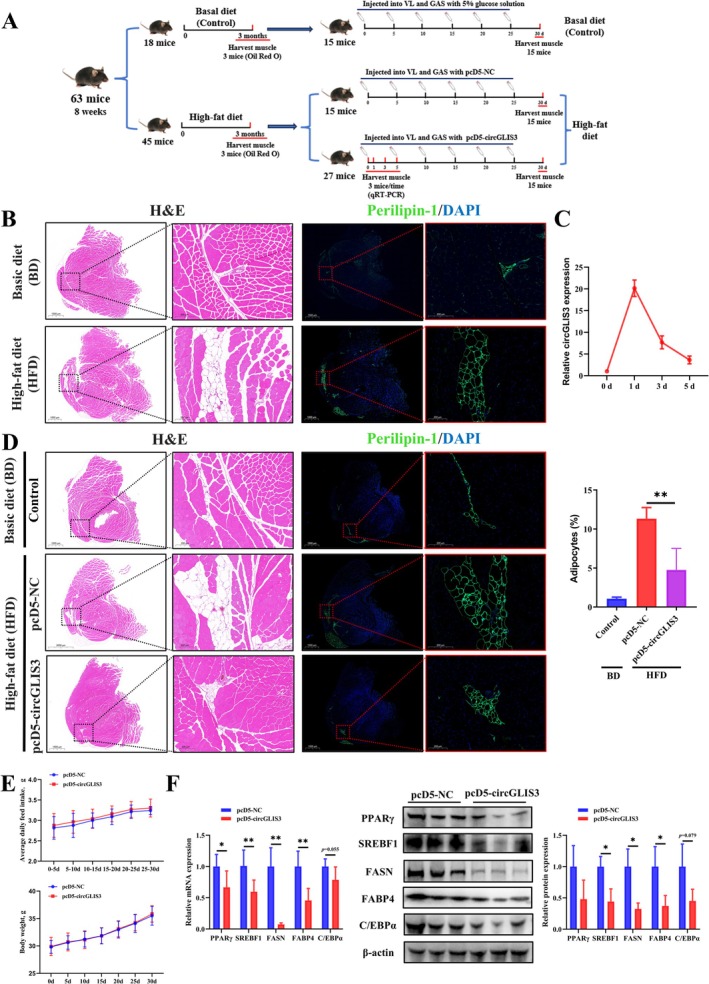
Overexpression of *circGLIS3* reduces skeletal muscle fat infiltration in mice. (A) Schematic diagram of the animal experimental procedure. (B) H&E staining and immunofluorescenc (adipocytes labelled with Perilipin‐1 [green], nuclei with DAPI [blue]) showing adipocyte infiltration in the VL muscles of mice after 3 months of high‐fat feeding. *n* = 3, scale bars 1000 and 200 μm. (C) After the *circGLIS3* expression plasmid (pcD5‐circGLIS3) was transfected into VL muscles, *circGLIS3* expression was detected by qRT‐PCR (*n* = 3) at 0, 1, 3 and 5 days. (D) After transfection with pcD5‐circGLIS3 in mouse muscle (VL), H&E staining and immunofluorescenc (adipocytes labelled with Perilipin‐1 [green], nuclei with DAPI [blue]) showing adipocyte infiltration in the VL of mice at 30 days. *n* = 6, scale bars 1000 and 200 μm. Quantification of the percentage (%) of adipocytes infiltrated areas in VL muscle tissue. (E) Average daily feed intake and body weight of mice at different time points after intramuscular injection of pcD5‐NC or pcD5‐circGLIS3 (*n* = 15). (F) After transfection with pcD5‐circGLIS3 in VL muscle, relative mRNA and protein expression levels of PPARγ, SREBF1, FASN, FABP4 and C/EBPα were analysed by qRT‐PCR (*n* = 6) and Western blot (*n* = 3) at 30 days. Results are presented as the means ± SD; *p < 0.05, **p < 0.01.

To further validate the effect of *circGLIS3* on fat infiltration in skeletal muscle, we designed a mouse injection assay, with the experimental procedure illustrated in Figure 5A. We collected VL muscle samples from mice fed either a basic diet (BD) or a HFD for 3 months and performed histology analysis. H&E staining results showed that the adipocyte infiltration area in the HFD group was significantly higher than that in the BD group. Immunostaining with Perilipin‐1, a well‐established adipocyte marker [23, 24], antibody further confirmed this result (Figure 5B). This result indicated that the HFD‐induced skeletal muscle fat infiltration model has been successfully established and can be used for subsequent experiments. The results of detecting *circGLIS3* overexpression efficiency in mouse muscle showed a slight decrease to just under a 5‐fold increase after 5 days of pcD5‐circGLIS3 injection (Figure 5C). To maintain the overexpression effect, we injected the plasmid every 5 days in the subsequent mouse injection experiment. After the injection trial, H&E staining results showed that *circGLIS3* overexpression significantly reduced the area of adipocyte infiltration in the skeletal muscle (Figure 5D). Immunostaining with Perilipin‐1 antibody further confirmed this result. The results of average daily feed intake (ADFI) and body weight (BW) assay showed that intramuscular injection of pcD5‐circGLIS3 did not affect the ADFI or BW of the mice (Figure 5E). This indicates that the reduction in adipocyte infiltration in muscle by *circGLIS3* is not directly influenced by food intake or body weight. Subsequently, we performed WB and qRT‐PCR experiments, which showed that *circGLIS3* overexpression significantly suppressed the mRNA and protein expression of SREBF1, FASN and FABP4 in mouse muscle (Figure 5F). These findings were consistent with results observed in primary bovine intramuscular preadipocytes and 3T3‐L1 cells. Together with the results from H&E staining and immunofluorescence, these data suggest that *circGLIS3* affects lipid metabolism in mouse intramuscular adipocytes, although the precise underlying mechanisms require further investigation. These findings suggest that *circGLIS3* alleviates HFD‐induced fat infiltration in mouse skeletal muscle.

### 3.6 *CircGLIS3* Regulates Adipogenesis in Primary Bovine Intramuscular Preadipocytes by Acting as a Sponge for miR‐21‐3p

**FIGURE 6:**
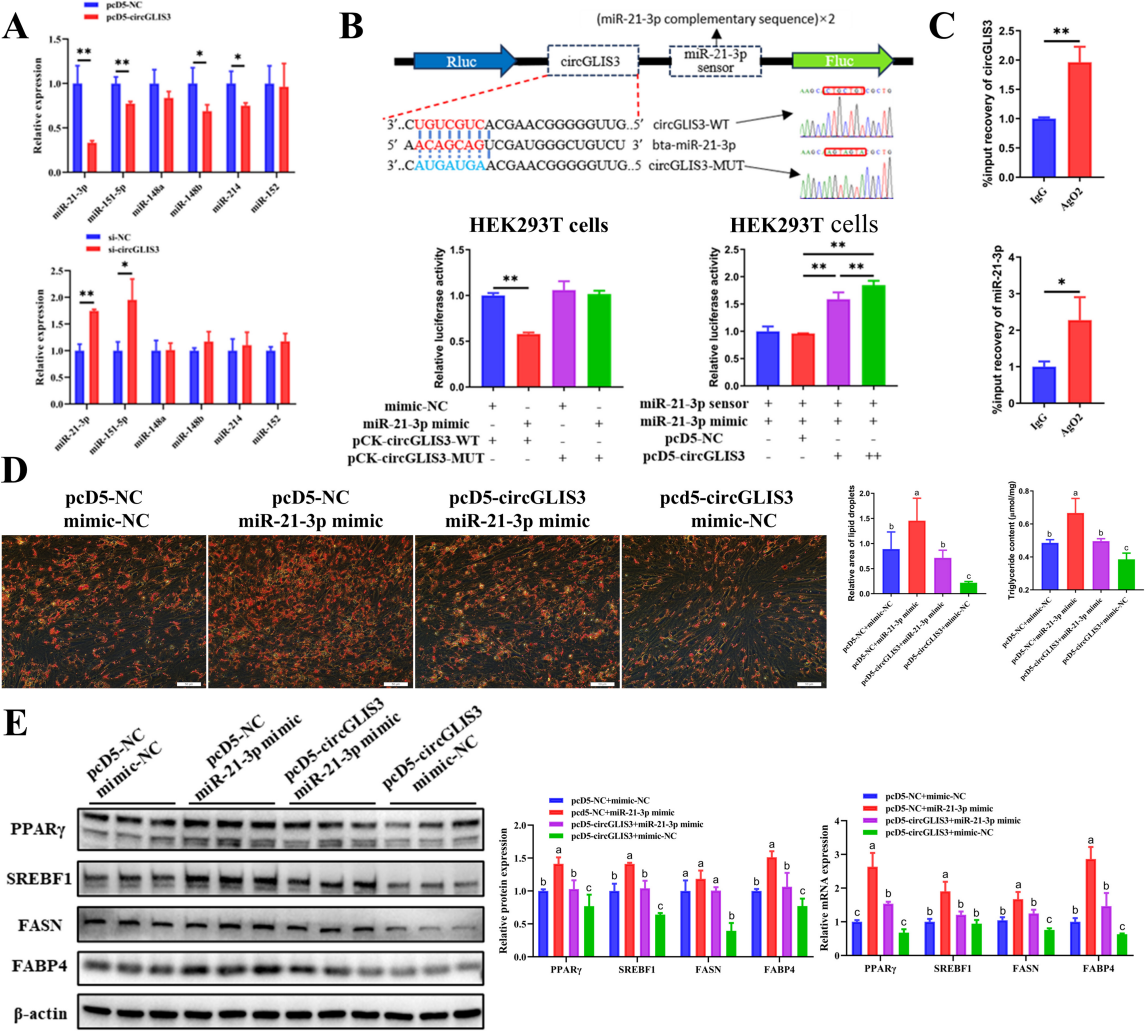
*CircGLIS3* regulates adipogenesis of by acting as a sponge for miR‐21‐3p. (A) The relative expression of the predicted target miRNAs of *circGLIS3* was detected by qRT‐PCR (*n* = 6) on the third day of differentiation after transfection with pcD5‐circGLIS3 or si‐circGLIS3 in primary bovine intramuscular adipogenesis. (B) Construction of luciferase reporter vectors. The miR‐21‐3p mimic was co‐transfected with psiCHECK2‐circGLIS3‐WT or psiCHECK2‐circGLIS3‐MUT into HEK293T cells, and luciferase activities were measured at 48 h post‐transfection (left, *n* = 3). The miR‐21‐3p biosensor (psiCHECK2‐miR‐21‐3p 2×) was co‐transfected with the miR‐21‐3p mimic and 1× or 2× pcD5‐circGLIS3 into HEK293T cells, and luciferase activities were measured at 48 h post‐transfection (right, *n* = 3). (C) After performing the RIP assay in primary bovine intramuscular adipogenesis using the AgO2 antibody, the enrichment levels of *circGLIS3* and miR‐21‐3p were quantified by qRT‐PCR (*n* = 3). (D) The pcD5‐circGLIS3 was co‐transfected with miR‐21‐3p mimic into primary bovine intramuscular preadipocytes, the lipid droplet content (determined by Oil Red O staining, *n* = 9, scale bar 50 μm) and triglyceride content (*n* = 3) were determined on the sixth day after differentiation. (E) The relative mRNA (*n* = 6) (M) and protein (*n* = 3) expression levels of PPARγ, SREBF1, FASN and FABP4 were analysed on the third day after differentiation. Results are presented as the means ± SD; *p < 0.05, **p < 0.01; different lowercase letters indicate significant differences (*p* < 0.05).

*CircGLIS3* were predominantly located in the cytoplasm (Figure 2E–F), whereas circRNAs located in the cytoplasm affect the expression of downstream target genes mainly through competitive adsorption of miRNAs [25, 26]. The miRNA binding capacity of *circGLIS3* was analysed using prediction tools and six miRNAs highly expressed in intramuscular preadipocytes were obtained by qRT‐PCR. Notably, both the interference and overexpression of *circGLIS3* significantly affected the expression of miR‐21‐3p and miR‐151‐5p (Figure 6A). Based on the predicted potential binding sites, luciferase reporter vectors were constructed (Figures 6B and S3A). The results of the dual‐luciferase reporter assay suggested that miR‐21‐3p significantly inhibited the Rluc activity of pCK‐circGLIS3‐WT in HEK293T cells (Figure 6B). However, the binding site of *circGLIS3* to miR‐151‐5p did not achieve the same effect (Figure S3A). The co‐transfection assay suggested that the overexpression of *circGLIS3* was able to compete with the miR‐21‐3p biosensor to bind miR‐21‐3p, which also resulted in remarkably promoted biosensor Rluc activity, and Rluc activity was significantly restored in a dose‐dependent manner (Figure 6B). A RIP experiment was performed using primary bovine intramuscular preadipocytes, and the results demonstrated that *circGLIS3* and miR‐21‐3p were significantly enriched in anti‐AgO2 (Figure 6C). Therefore, miR‐21‐3p was selected as the target miRNA of *circGLIS3* for subsequent experiments.

The loss‐of‐function experimental results demonstrated that miR‐21‐3p knockdown inhibited adipogenesis in primary bovine intramuscular preadipocytes (Figure S3B–D), consistent with the effects observed in *circGLIS3* overexpression experiments. We transfected varying doses of pcD5‐circGLIS3 into intramuscular preadipocytes under miR‐21‐3p knockdown conditions. The results showed that in miR‐21‐3p‐intact intramuscular preadipocytes, adipogenic differentiation ability decreased progressively with increasing *circGLIS3* overexpression. However, in miR‐21‐3p knockdown intramuscular preadipocytes, no significant changes in adipogenic differentiation were observed (Figure S3E–F). These findings suggest that miR‐21‐3p knockdown attenuates the ability of *circGLIS3* to inhibit adipogenic differentiation in intramuscular preadipocytes via the miR‐21‐3p sponge mechanism. The results of the rescue experiment showed that overexpression of miR‐21‐3p effectively alleviated the inhibitory effect of *circGLIS3* overexpression on adipogenic differentiation (Figure 6D–E), including lipid droplet accumulation, triglyceride synthesis, and the expression of differentiation‐related markers. Overall, these studies suggest that *circGLIS3* inhibits primary bovine intramuscular adipogenesis by sponging miR‐21‐3p.

### 3.7 *CircGLIS3* Upregulates *LEPR* Expression by Sponging miR‐21‐3p, Thereby Inhibiting the Adipogenic Differentiation of Primary Bovine Intramuscular Preadipocytes

**FIGURE 7:**
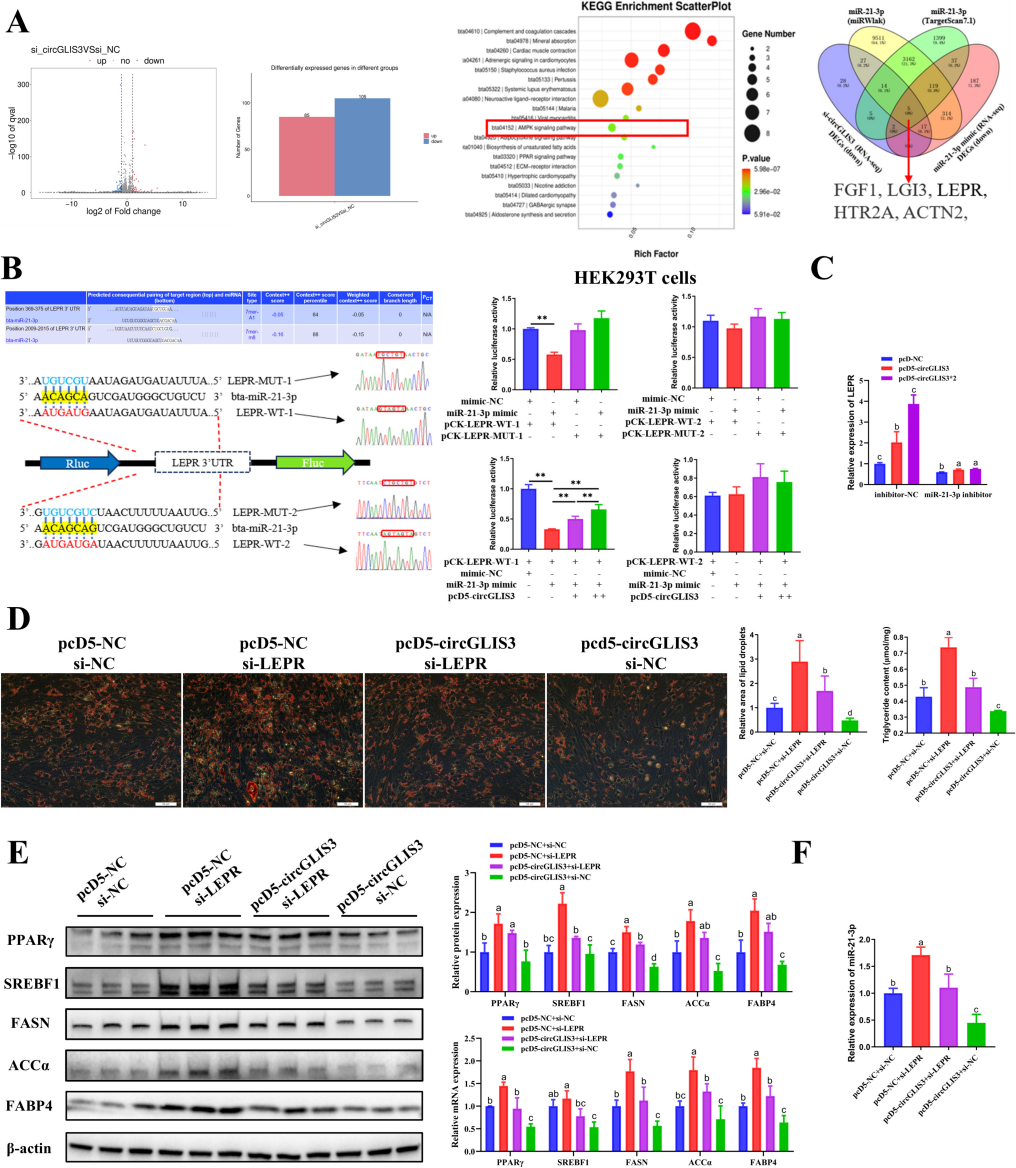
*CircGLIS3* functions as a ceRNA to regulate *LEPR* expression by sponging miR‐21‐3p. (A) Transcriptome analysis was performed on the third day of differentiation after transfection with si‐circGLIS3 in primary bovine intramuscular preadipocytes. Volcano plots and histograms display DEGs, and KEGG pathway enrichment analysis was conducted. Overlap analysis of target genes predicted by miRWlak and TargetScan7.1 platforms with the downregulated DEGs (identified by RNA‐seq on the third day of differentiation after transfection with si‐circGLIS3 or the miR‐21‐3p mimic). (B) TargetScan software predicts the binding site of miR‐21‐3p to the target gene *LEPR* and luciferase reporter vectors. miR‐21‐3p mimic was co‐transfected with pCK‐LEPR‐WT‐1/2 or pCK‐LEPR‐MUT‐1/2 into HEK293T cells, and luciferase activities were measured at 48 h post‐transfection (*n* = 3). The pCK‐LEPR‐WT‐1/2 were co‐transfected with the miR‐21‐3p mimic, or 1× or 2× pcD5‐circGLIS3 into HEK293T cells, and luciferase activities were measured at 48 h post‐transfection (*n* = 3). (C) Under miR‐21‐3p knockdown conditions, primary bovine intramuscular preadipocytes were transfected with varying doses of pcD5‐circGLIS3, relative mRNA expression levels of *LEPR* were analysed on the third day after differentiation (*n* = 6). (D) The pcD5‐circGLIS3 was co‐transfected with si‐LEPR into primary bovine intramuscular preadipocytes, lipid droplet content (determined by Oil Red O staining, *n* = 9, scale bar 50 μm) and triglyceride content (*n* = 3) were determined on the sixth day after differentiation. (E) The relative protein (*n* = 3) and mRNA (*n* = 6) expression levels of PPARγ, SREBF1, FASN, ACCα and FABP4 were analysed on the third day after differentiation. (F) The pcD5‐circGLIS3 was co‐transfected with the si‐LEPR into primary bovine intramuscular preadipocytes, and relative expression levels of miR‐21‐3p were analysed on the third day after differentiation (*n* = 6). Results are presented as the means ± SD; *p < 0.05, **p < 0.01; different lowercase letters indicate significant differences (*p* < 0.05).

Having identified the miRNA that *circGLIS3* can adsorb in the regulation of intramuscular lipogenesis, the next step is to identify the target genes that competitively adsorb miR‐21‐3p with *circGLIS3*. RNA‐seq was performed on the third day of differentiation following transfection with si‐circGLIS3. Inter‐sample correlation analysis on the raw data revealed a high correlation between samples within the group (Figure S4A–C). Compared to the control group, transfection with si‐circGLIS3 resulted in 85 DEGs upregulated and 105 DEGs downregulated (Figure 7A). KEGG pathway enrichment analysis showed that the pathways associated with adipogenesis included the AMPK, adipocytokine and PPAR signalling pathways (Figure 7A, Additional file 8). In addition, by overlapping the predicted target genes of miR‐21‐3p with the downregulated DEGs identified by RNA‐seq on the third day of differentiation after transfection with si‐circGLIS3 or the miR‐21‐3p mimic, five candidate targets were screened (Figure 7A, Additional file 9). Notably, *LEPR* is a key gene in the AMPK signalling pathway. We found that *circGLIS3* overexpression alleviated the inhibitory effect of miR‐21‐3p on *LEPR* expression (Figure S4D). Based on two potential binding sites of *LEPR* with miR‐21‐3p, luciferase reporter vectors were successfully constructed (Figure 7B). The results of the dual‐luciferase reporter assay indicated that miR‐21‐3p significantly inhibited the Rluc activity of pCK‐LEPR‐WT‐1 in HEK293T cells, but had no effect on pCK‐LEPR‐MUT‐1, pCK‐LEPR‐WT‐2 or pCK‐LEPR‐MUT‐2 (Figure 7B). Subsequent co‐transfection experiments revealed that *circGLIS3* overexpression counteracted the suppressive effect of miR‐21‐3p on Rluc activity in a dose‐dependent manner (Figure 7B). These results suggested that *circGLIS3* functions as a ceRNA to regulate *LEPR* by sponging miR‐21‐3p. We transfected varying doses of pcD5‐circGLIS3 into primary bovine intramuscular preadipocytes under miR‐21‐3p knockdown conditions. The results showed that, in miR‐21‐3p‐intact intramuscular preadipocytes, *LEPR* expression increased with increasing *circGLIS3* overexpression, but no significant changes were observed in miR‐21‐3p knockdown intramuscular preadipocytes (Figure 7C). These findings suggest that miR‐21‐3p knockdown diminishes the ability of *circGLIS3* to regulate *LEPR* expression via the miR‐21‐3p sponge mechanism.

Gain‐of‐function and loss‐of‐function experiment results showed that *LEPR* inhibited adipogenesis in primary bovine intramuscular preadipocytes, consistent with the results of *circGLIS3* (Figure S5A–F). In the ceRNA mechanism of ncRNAs, circRNAs interacts with its target genes by adsorbing miRNAs, while the target genes themselves function as the ultimate effectors responsible for executing the biological functions. The results of the rescue experiment showed that si‐LEPR effectively alleviated the inhibitory effect of *circGLIS3* overexpression on adipogenic differentiation of intramuscular preadipocytes (Figure 7D–E), including lipid droplet accumulation, triglyceride synthesis, and the expression of differentiation‐related markers. Furthermore, *circGLIS3* overexpression also attenuated the promoting effect of si‐LEPR on adipogenic differentiation. In the co‐transfection experiment of pcD5‐circGLIS3 and si‐LEPR, both treatments were found to alleviate the regulatory effect of the other on miR‐21‐3p expression (Figure 7F). Overall, we determined that *circGLIS3* competes with *LEPR* to sponge miR‐21‐3p, thereby promoting *LEPR* expression and inhibiting adipogenic differentiation of intramuscular preadipocytes.

### 3.8 *CircGLIS3* Upregulates *LEPR* Expression by Sponging miR‐21‐3p, Thus Activating the AMPK Signalling Pathway

**FIGURE 8:**
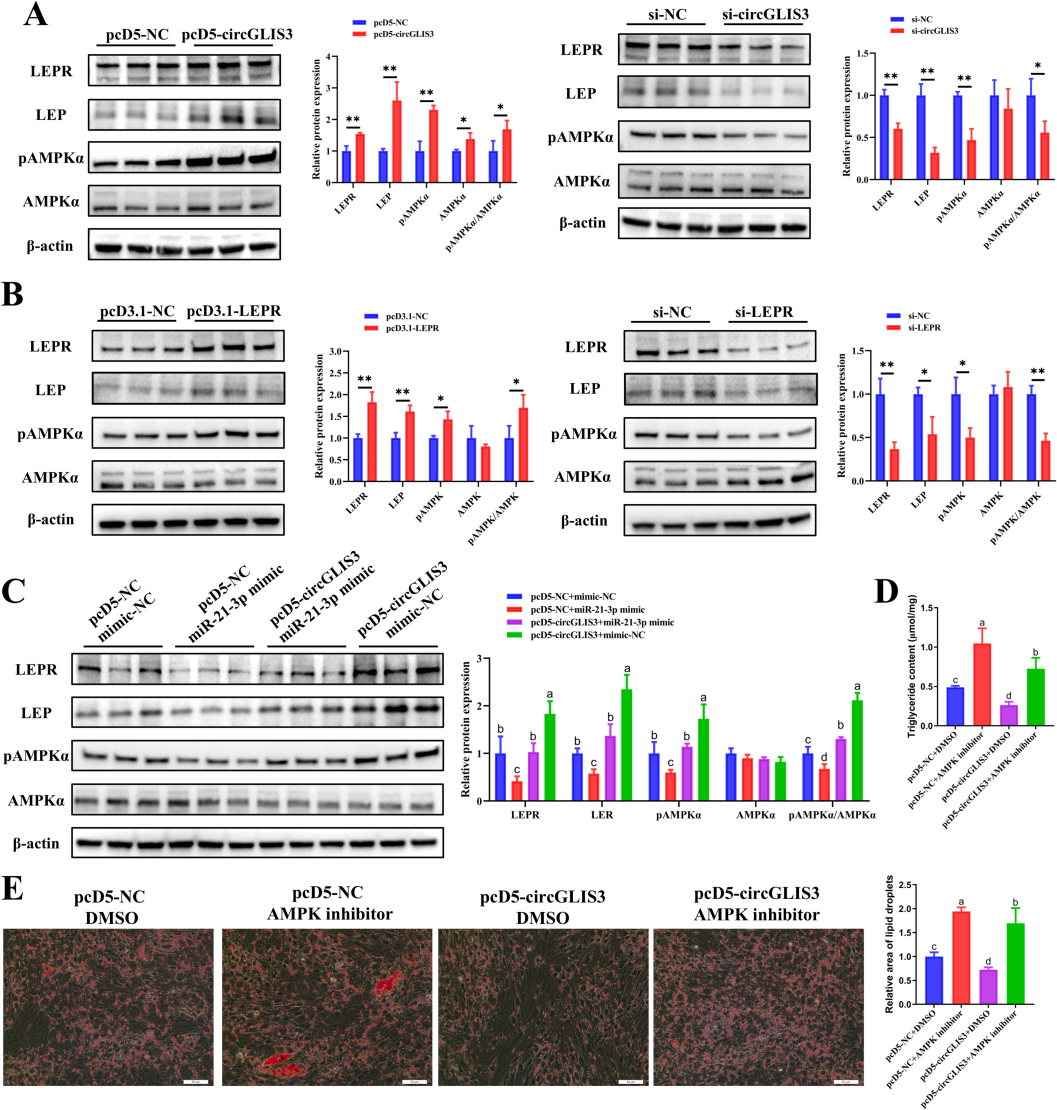
The miR‐21‐3p/LEPR/AMPK axis is required for the function of *circGLIS3* in primary bovine intramuscular adipogenesis. (A,B) Relative protein expression levels of LEPR, LEP, AMPKα and p‐AMPKα were analysed (*n* = 3) on the third day after differentiation after transfection with pcD5‐circGLIS3, si‐circGLIS3 (A), pcD3.1‐LEPR or si‐LEPR (B). (C) *CircGLIS3* was co‐transfected with the miR‐21‐3p mimic into intramuscular preadipocytes, and the relative protein expression levels of LEPR, LEP, AMPKα, and p‐AMPKα were analysed (*n* = 3) on the third day after differentiation. (D,E) Triglyceride content (*n* = 3) and lipid droplet content (determined by Oil Red O staining, *n* = 9, scale bar 50 μm) were determined on the sixth day of differentiation following treatment with AMPK inhibitor (5 μM) and/or pcD5‐circGLIS3. Results are presented as the means ± SD; *p < 0.05, **p < 0.01; different lowercase letters indicate significant differences (*p* < 0.05).

KEGG enrichment analysis of RNA‐seq data from differentiated intramuscular preadipocytes following *circGLIS3* interference revealed significant enrichment of the AMPK pathway, which is associated with adipogenesis. The results of this experiment showed that overexpression of *circGLIS3* and *LEPR* in primary bovine intramuscular preadipocytes significantly increased the protein expression levels of LEPR, LEP and phospho‐AMPKα (pAMPKα), as well as the pAMPKα/AMPKα ratio, whereas interference with *circGLIS3* and *LEPR* had opposite effects (Figure 8A,B). Additionally, co‐transfection assays indicated that miR‐21‐3p mimic inhibited the upregulation of LEPR, LEP and pAMPKα protein expression induced by *circGLIS3* overexpression (Figure 8C). We overexpressed *circGLIS3* and/or treated them with 5 μM compound C (a selective AMPK inhibitor) [27] in intramuscular preadipocytes; results showed that the AMPK inhibitor alleviates the inhibitory effect of *circGLIS3* overexpression on lipid droplet formation and triglyceride synthesis (Figure 8D,E). This result suggests that *circGLIS3* inhibits intramuscular preadipocyte adipogenic differentiation through the AMPK signalling pathway. Based on all our findings, we propose a *circGLIS3*‐mediated regulatory model of intramuscular adipogenesis. *CircGLIS3* acts as a decoy for miR‐21‐3p, alleviating its inhibitory effect on *LEPR* and activating the AMPK signalling pathway to inhibit the adipogenic differentiation of primary bovine intramuscular preadipocytes.

## 4 Discussion

Muscle mass accounts for approximately 40% of the body weight in mammals [28]. In meat production, abundant IMF deposition enhances meat flavour and nutritional value. However, in human skeletal muscle, excessive IMF deposition, referred to as fat infiltration or myosteatosis, is a common hallmark of chronic skeletal muscle injury and disease. Intramuscular adipogenesis is a complex process, and its regulatory mechanisms remain poorly understood. Studies have indicated a direct relationship between intramuscular adipogenesis and FAPs, which generate preadipocytes and myoblasts under specific conditions [29]. Studies have shown that bovine FAPs are more similar to human FAPs compared to those of mice [18]. This study explored the role of *circGLIS3* in regulating intramuscular adipogenesis and reducing muscle fat infiltration through in vitro experiments with bovine intramuscular preadipocytes and in vivo experiments in obese mice.

CircRNAs were first identified in plant viroids in 1976 [30]. Since then, a substantial number of circRNAs have been discovered in humans, animals and plants. Some of these circRNAs have been shown to be involved in the pleiotropic regulation of cellular functions and play critical regulatory roles in muscle and fat development [10, 15, 17]. Although intensive studies have confirmed the role of circRNAs in muscle and fat development or diseases, few studies have investigated the relationship between circRNAs and IMF deposits. In this study, we screened *circGLIS3*, a circRNA closely associated with intramuscular adipogenesis, through circRNA‐seq and WGCNA. Previous studies have shown that research on multiple *circGLIS3* isoforms derived from the *GLIS3* gene has primarily focused on humans and mice. These *circGLIS3* isoforms are closely associated with obesity‐induced β‐cell dysfunction and the progression of various cancers, including kidney, prostate, thyroid, and bladder cancers. Each *circGLIS3* isoform exerts its effects through specific regulatory mechanisms, such as interactions with miRNAs, protein binding, or the encoding of small peptides (Table S7) [31, 32]. This indicates that *circGLIS3* has diverse biological functions across different tissues and diseases. Notably, *circGLIS3* exhibits high homology among bovine, mouse and human species. Our results suggest that *circGLIS3* inhibits adipogenesis in bovine intramuscular preadipocytes and 3T3‐L1 cells while also mitigating HFD‐induced intramuscular fat infiltration in mice. These findings suggest that *circGLIS3* not only serves as a promising target for improving meat quality in livestock but also as a potential therapeutic marker for alleviating skeletal muscle fat infiltration associated with obesity.

The best‐known functional pattern of circRNAs is their ability to serve as miRNA ‘sponges’. MiRNAs are a class of endogenous small noncoding RNAs that silence target gene expression primarily through interactions with the 3′ untranslated region (3′UTR) of target mRNAs [33]. Our results indicate that circGLIS3 acts as a decoy for miR‐21‐3p through the ceRNA mechanism, influencing the expression of *LEPR*. However, the role of miR‐21‐3p in adipogenic differentiation remains largely unexplored. Only one study has shown that miR‐21‐3p promotes triglyceride production in mammary epithelial cells of dairy cows [34]. Therefore, this study confirmed that miR‐21‐3p knockdown inhibited adipogenic differentiation of intramuscular preadipocytes. Leptin (LEP), produced by adipocytes, regulates obesity in animals by interacting with LEPR in hypothalamic neurons [35]. However, the direct effects of LEP/LEPR signalling on adipocytes at the cellular level have been controversial. Our previous research has confirmed that *LEP* inhibits bovine intramuscular adipogenesis through the LEPR/AMPK signalling pathway [27]. This study also demonstrated that *LEPR* inhibits adipogenesis in bovine intramuscular preadipocytes. Notably, KEGG enrichment analysis of the DEGs identified by RNA‐seq of intramuscular preadipocytes treated with si‐circGLIS3 revealed enrichment of the AMPK signalling pathway. AMPK is well known as a crucial regulator of various biological functions in skeletal muscle, including muscle atrophy, lipid metabolism, and mitochondrial function [36]. It has been shown that LEP/LEPR stimulates FA oxidation by activating the AMPK signalling pathway [37]. Our results indicate that *circGLIS3* acts as a decoy for miR‐21‐3p, alleviating its inhibitory effect on *LEPR* and activating the AMPK signalling pathway to inhibit adipogenic differentiation of primary bovine intramuscular preadipocytes. Previous studies have shown that the AMPK signalling pathway inhibits de novo lipid synthesis by regulating the expression of the transcription factor SREBF1 and its downstream rate‐limiting enzymes, including FASN, ACCα and FABP4 [38]. Our results suggest that the circGLIS3/miR‐21‐3p/LEPR axis significantly affects the expression of SREBF1, FASN, ACCα, and FABP4. Therefore, we conclude that *circGLIS3* acts as a sponge for miR‐21‐3p to inhibit SREBF1 as well as its downstream FASN, ACCα, and FABP4 expression by activating the LEPR/AMPK signalling pathway, thereby effectively suppressing intramuscular adipogenesis.

The key factors determining IMF deposition are the number of intramuscular preadipocytes and their adipogenic capacity [39]. The maturation and differentiation of preadipocytes affect their adipogenic ability, while proliferation and apoptosis determine their quantity [40]. This study primarily investigates the effect and regulatory mechanisms of *circGLIS3* on adipogenic differentiation in intramuscular preadipocytes. However, further research on the effects of *circGLIS3* on proliferation and apoptosis is needed to fully understand the impact and mechanisms of *circGLIS3* on IMF deposition. Additionally, the in vivo experiments in this study only examined the role of *circGLIS3* in alleviating skeletal muscle fat infiltration in mice, without exploring its specific in vivo regulatory mechanisms, which also require further investigation.

## 5 Conclusion

This study identified a novel circular RNA, *circGLIS3*, and verified its role and regulatory mechanism in intramuscular adipogenesis. Mechanistically, *circGLIS3* acts as a sponge for miR‐21‐3p to inhibit SREBF1 as well as its downstream FASN, ACCα and FABP4 expression by activating the LEPR/AMPK signalling pathway, thereby effectively suppressing intramuscular adipogenesis. In summary, these findings provide novel insights into the regulatory mechanisms of intramuscular adipogenesis and theoretically support *circGLIS3* as a potential target for improving beef quality through directional selection and molecular breeding. Moreover, in vivo studies conducted in a mouse model provide evidence supporting the potential application of *circGLIS3* in treating human obesity‐related skeletal muscle inflammation.

## Consent

The authors have nothing to report.

## Conflicts of Interest

The authors declare no conflicts of interest.

## Supporting Information

Additional supporting information may be found online in the Supporting Information section at the end of the article.

## Supporting information


**Table S1.** The sequence information utilized in this study.
**Table S2.** Oligonucleotide sequences in this study.
**Table S3.** Primers utilized in this study.
**Table S4.** Primer information of miRNAs utilized in this study.
**Table S5.** The sequence information of RNA‐FISH.
**Table S6.** The information on antibodies.
**Table S7.** Research progress on circRNAs derived from the GLIS3 gene and their homology comparison with bovine *circGLIS3*.

**Material S1.** The details of the supplementary materials and methods are provided.

**Additional file 1.** The differentially expressed circRNAs (DECs) identified at four stages of adipogenic differentiation (0, 3, 6 and 9 days post‐differentiation).

**Additional file 2.** KEGG pathway analysis of the parental genes associated with DECs.

**Additional file 3.** Module information of WGCNA.

**Additional file 4.** Homology analysis.

**Additional file 5.** Homology analysis.

**Additional file 6.** Homology analysis.

**Additional file 7.** Homology analysis.

**Additional file 8.** KEGG pathway analysis of differentially expressed genes.

**Additional file 9.** Prediction of the target genes of miR‐21‐3p.

**Figure S1.** (A) KEGG pathway analysis. (B) Hierarchical clustering tree of all samples. (C) Determination of soft‐thresholding power. When the optimal soft‐threshold was chosen 8, with *R* ^2^ = 0.85 and mean connectivity < 100, the network was scale‐free topology. (D) Hierarchical cluster analysis of different modules. The red line represents a cut height of 0.5 to merge modules with more than 50% similarity. (E) Hierarchical clustering of genes with dissimilarity based on topological overlap is shown in the detected and merged modules.

**Figure S2.** (A–F) Sanger sequencing of candidate circRNAs confirmed the back‐splicing junction sequence (above), and qRT‐PCR (*n* = 6) detected the expression of candidate circRNAs in different developmental stages of primary bovine intramuscular preadipocytes (below).

**Figure S3.** (A) Bioinformatics analysis was used to predict the binding between circGLIS3 and miR‐21‐3p or miR‐151‐5p. The construction of the luciferase reporter vector and related experiments were performed using HEK293T cells (*n* = 3). (B) After transfecting miR‐21‐3p mimic or inhibitor into primary bovine intramuscular preadipocytes, the expression efficiency of miR‐21‐3p interference and overexpression was determined by qRT‐PCR (*n* = 6) on the third day of differentiation. (C) Lipid droplet content (determined by Oil Red O staining, *n* = 9, scale bar 50 μm) and triglyceride content (*n* = 3) were determined on the sixth day of differentiation in primary bovine intramuscular preadipocytes. (D) Relative mRNA and protein expression levels of PPARγ, SREBF1, FASN and FABP4 were analysed by qRT‐PCR (*n* = 6) and Western blot (*n* = 3) on the third day of differentiation in primary bovine intramuscular preadipocytes. (E) Under miR‐21‐3p knockdown conditions, primary bovine intramuscular preadipocytes were transfected with varying doses of pcD5‐circGLIS3, the expression of *circGLIS3* was determined by qRT‐PCR (*n* = 6) on the third day of differentiation. (F) Under miR‐21‐3p knockdown conditions, primary bovine intramuscular preadipocytes were transfected with varying doses of pcD5‐circGLIS3, triglyceride content (*n* = 3) and the lipid droplet content (determined by Oil Red O staining, *n* = 9, scale bar 50 μm) were determined on the sixth day of differentiation. Results are presented as the means ± SD, *p < 0.05; **p < 0.01; different lowercase letters indicate significant differences (*p* < 0.05).

**Figure S4.** Transcriptome analysis was performed on the third day of differentiation after transfection with si‐circGLIS3 in primary bovine intramuscular preadipocytes. (A–C) Correlation analysis between samples. (D) The pcD5‐circGLIS3 was co‐transfected with the miR‐21‐3p mimic into intramuscular preadipocytes, and relative mRNA expression levels of *LEPR* were analysed on the third day after differentiation (*n* = 6) (below). Results are presented as the means ± SD, different lowercase letters indicate significant differences (*p* < 0.05).

**Figure S5.** *LEPR* inhibits adipogenesis of primary bovine intramuscular preadipocytes, and *circGLIS3* overexpression reverses the adipogenesis promotion induced by si‐LEPR. (A) qRT‐PCR (*n* = 3) detected the expression of LEPR on the third day of differentiation after transfection with pcD3.1‐LEPR or si‐LEPR. (B,C) Oil Red O staining (*n* = 9, scale bar 50 μm) evaluated the lipid droplet content of intramuscular adipocytes on the sixth day of differentiation. (D) Determination of triglyceride content (*n* = 3) on the sixth day of differentiation after transfection with pcD3.1‐LEPR or si‐LEPR. (E,F) Relative mRNA and protein expression levels of PPARγ, SREBF1, FASN, FABP4 and C/EBPα were analysed by qRT‐PCR (*n* = 6) and Western blot (*n* = 3) on the third day of differentiation. Results are presented as the means ± SD, *p < 0.05; **p < 0.01.

## Data Availability

The data that support the findings of this study are available in the main manuscript and supplemental materials. The RNA‐seq raw data reported in this paper have been deposited in the Genome Sequence Archive (Genomics, Proteomics and Bioinformatics 2021) in the China National Center for Bio‐information. It can be accessed through the GSA series numbers CRA017448 (si‐circGLIS3) and CRA017451 (miR‐21‐3p mimic).
