# Supplementary material for: CircGLIS3 Inhibits Intramuscular Adipogenesis and Alleviates Skeletal Muscle Fat Infiltration
Source: J Cachexia Sarcopenia Muscle. 2025 Jul 30;16(4):e70009. doi: 10.1002/jcsm.70009 (PMC12308225; doi:10.1002/jcsm.70009)
Supplement: Supplementary file 1 — Table S1. The sequence information utilized in this study. Table S2. Oligonucleotide sequences in this study. Table S3. Primers utilized in this study. Table S4. Primer information of miRNAs utilized in this study. Table S5. The sequence information of RNA‐FISH. Table S6. The information on antibodies. Table S7. Research progress on circRNAs derived from the GLIS3 gene and their homology comparison with bovine circGLIS3. [file JCSM-16-e70009-s014.docx]

**Supplementary Tables**

**Table S1.** The sequence information utilized in this study.

| **Name** | **Sequences (5′-3′)** |
| --- | --- |
| circGLIS3  (circRNA32) | GGGCTCTTGGCTTTGGGCCTCAGTGCAAGTCCATTGGAAAAGGCAGCTGCAACAATCTAGTGGTCACCAGCAGTCCCATGATGGTTCAGCGACTGGGACCCATTTCACCTCCAGCAAGCCAGGTCTCTACAGCATGCAACCAGATCAGTCCTAGCTTACAGAGGGCAATGAATGCAGCCAACCTGAATATACCTCCTTCAGATACCAGGTCCCTTATTTCACGAGAGTCTTTGGCCTCCTCAAATTTGAGTCTGACAGAAAGTCAGTCGACCTTGAACGTGAAACAAGAGTGGTCGCACGGCTATAGGGCTCTCCCTTCGCTCTCCTCGAACCACAACTCTCAGAATGGCACTGATCTAGGGGACCTAATTAGCCTTCCTCCTGGGACATCCATGTCCAGCAACAGTGTCTCTAACTCATTGCCACCCTACCTTTTCGGTATGGAAAATAGCCACTCTCCTTACCCCAGTCCCCGACACTCTTCAGCCAGGTCCCACTCGGCCCGCTCCAAGAAGAGAGCACTGTCCTTGTCCCCGTTGTCCGATGGCATCGGGATAGACTTCAATACCATCATCCGCACCTCGCCCACATCCTTGGTCGCCTACATCAACGGTTCGAGGGCTTCCCCCGCCAACATGTCTCCACAGCCTGAGGTCTACGGGCATTTCCTGGGAGTGCGAGGCAGCTGTATTCCCCAGCCATGCTCAATGCCAAGCAGCCAAAAGGGTGTGCGGGTGGCCAGCGGCGGCCTGGCTCTCCCGGCCTACGATGAGGACGGTGCACTGGAGTATGAGCGCATGCAGCAGCTGGAGCACGGCGGCCTCCAACCCGGCCTGGTCAACAACATGGTGGTGCAGCACGGCCTGCCGGACCCTGCCGGCCACGCGGCCGGCCTGCTGAAGACGGAGCGCCTGGATGACTTCCCTGGTAGCGTCCTGGACCTGCCCCCCGCACCCTCTCTGCCTCCTCTGCCACCGCCGCCCCAGCCCCAAGGCCCCCCGCCACCCTATCATGCCCACCCGCATCTGCATCACCCAGAGCTGGTCCACCAGGCCCAGCCGCTGGCCCTGCCTCAGGCCGCCCTGGAGGAGGATGGGGAGATGGATGACGTTGGGGGCAAGCACTGCTGTCGCTGGATCGACTGCAGTGCCTTGTATGACCAGCAGGAGGAACTTGTGCGGCACATCGAGAAGGTTCATATAGACCAGCGCAAAGGCGAAGACTTCACTTGTTTCTGGGCCGGTTGTCCTCGAAGGTACAAGCCATTTAATGCCCGCTATAAACTGTTGATCCACATGAGAGTTCACTCGGGGGAAAAGCCCAACAAGTGTTCG |
| circRNA1057 | ATGATGATGTACCTGCAGATATGGTTGCAGAAGAATCCGGTCCTGGTGCACAAAATAGTCCATACCAACTTCGTAGGAAAACTCTTTTGCCAAAAAGAACAGCGTGTCCTACCAAGAGCAGTATGGAGGGTGCTTCAACTTCAACTACAGAAAACTTTTTTGGTCATCGTGCAAAACGTGCAAGAGTTTCTGGAAAATCACAAGATCTATCAGCACCTGCTGAACAGTATCTTCAGGAGAAACTGCCAGATGAAGTGGTTCTAAAAATCTTCTCGTATTTGCTGGAACAGGATCTTTGTAGAGCAGCTTGTGTGTGTAAACGCTTCAGTGAACTTGCTAATGATCCAATTTTATG |
| circRNA1290 | ATTCTTTCTAAAGTTTTTCCTCAAGTGCAATCAGAACTGTTTGAAGAATGCAGGCAACCCCCGGGACATGCGAAGATTCCAGGTTGTCGTGTCCACCACGGTCAACGTGGACGGCCATGTGCTGGCCGTGTCCGACAACATGTTTGTGCACAACAACTCCAAGCACGGGCGGCGGGCGCGCCGCCTGGACCCGTCAGAAGCCACTCCGTGCATCAAGGCCATCAGTCCCAGTGAAGGCTGGACCACGGGCGGCGCGACCGTGATCATCATTGGAGACAACTTCTTCGACGGGCTTCAAGTCGTGTTTGGGACGATGTTGGTGTGGAGTGAGCTGATCACGCCCCACGCCATCCGTGTCCAGACCCCACCGAGGCACATTCCTGGAGTCGTCGAAGTCACCTTGTCCTACAAGTCCAAGCAGTTCTGTAAAGGTGCTCCAGGGCGGTTTGTCTACACCG |
| circRNA1708 | GAGTACAAACCACCAGGAACCCGTAAACTGCATAATATTCTCGGAGTAGAAACAGGAGGGCCTGGTGGGCGGCGTGCTGGGGAGTCGGGCCATACAGTAGCTGACTACTTGAAGTTCAAAGACCTCATTTTAAGGATGCTTGACTATGACCCCAAATCTCGAATTCAACCTTACTATGCCCTGCAGCACAGTTTTTTCAAGAAAACAGCTGATGAAGGTACAAATACGAGTAATAGTGTATCCACGAGTCCTGCTATGGAACAGTCACAGTCTTCAGGCACCACCTCCAGTACATCTTCAAGCTCAGGTGGATCGTCGGGGACGAGCAACAGTGGGAGAGCCAGGTCGGACCCAACGCACCAGCATCGGCACAGCGGCGGGCACTTCACGGCCGCCGTGCAGGCCATGGACTGTGAGACCCACAGTCCGCAG |
| circRNA2209 | GCAGAAAAAACAATTGGCCACCTCTTCCTGACAACTTTCCTGTGGGCCCTTGTTTTTATCAGGATTTTTCTGTAGATATCCCTGTAGAATTCCAAAAAACAGTAAAGATTATGTACTACTTATGGATGTTCCATGCTGTAACACTATTTCTAAATATCTTCGGATGCTTGGCTTGGTTTTGTGTTGATCCTCCAAGAGGGGTTGATTTTGGATTGAGTATCCTGTGGTTCTTACTTTTTACTCCTTGTTCATTTGTCTGTTGGTACAGACCACTTTACGGAGCTTTCAGGAGTGACAGCTCCTTCCGGTTCTTCGTGTTCTTTTTTGTCTATATCTGTCAGTTTGCTGTGCACGTACTCCAGGCTGCAGGATTTCATAACTGGGGTAACTGTGGTTGGATTTCATCCCTTACTGGTCTCAACAAAAGTATTCCTGTTGGAATCATGATGATTATCATAGCAGCACTTTTCACAGCATCAGCAGTCATCTCACTAGTTATGTTTAAAAAG |
| circRNA2627 | GATTATAGTCATTGTAGTGAGGAAAGCAAAGGAAGTCAAAGCCATCCCAACTGGAAATGAAGAAGGAACTGCTTGCGTATGTAGAACAAAAAAAGTCTACTAACTTTGTGGTGAATTTTTGGATGAAGCCATTAAATTAATTGCTTGCCACCATGAGCAGAAGCAAGCGTGACAGCAATTTTTATAGTGTAGAGATTGGAGATTCTACATTCACAGTCCTGAAACGGTATCAGAATTTAAAACCTATAGGTTCAGGAGCCCAAGGAATAGTATGTGCAGCTTATGATGCCATTCTTGAAAGAAACGTTGCAATCAAGAAGTTAAGTCGGCCATTTCAGAATCAAACTCATGCTAAGCGTGCTTACAGAGAGCTAGTTCTCATGAAATGTGTAAATCACAAAAATATAATTGGCCTTTTGAATGTTTTTACACCACAGAAATCCCTAGAAGAATTTCAAGATGTTTACATAGTCATGGAGCTCATGGATGCAAATCTTTGTCAAGTGATTCAGATGGAGCTAGATCATGAAAGAATGTCCTACCTGCTCTATCAGATGCTGTGTGGGATCAAGCACCTTCACTCTGCTGGAATTATTCACCGG |
| circRNA4491 | ATTCTATCACAAGGTGCTATCTTTACATGAAGATTCAGCAACCCCTGTGTCTAACCCTCTGCTTGCATTTACTCTCATCAAACGCCTACAGTCTGACTGGAAGAATGTGGTACATAGTCTGGAGGCCAGTGAGAACATCCGAGCTCTCAAGGATGGTTATGAGAGGGTGGAGCAGGACCTGCCAGCCTTTGAGGACCTCGAGGGAGCAGCAAGGGCCCTGATGCGGCTGCAGGACGTGTACATGCTCAATGTGAAGGGACTTGCCCGAGGCGTCTTCCAGAGAGTCACAGGCTCTGCTGTCACTGACTTGTATAGTCCCAGGCGACTTTTCTCCCTCACCGGGGATGACTGCTTCCAAGTTGGCAAG |
| circRNA5655 | GAGTGTGAGGAGGAGGCTGTCGGGGTCATTATGTGCGCGTCGGTCAAGTACAACATCCGGGGTCCCGCCCTCATCCCGAGAATGAAGACCAAGCACCGCATCTACTACATCACCCTCTTCTCCATCGTCCTGCTGGGTCTGATCGCCACGGGCATGTTTCAGTTCTGGCCGCACTCCATCGAGTCCTCCGGCGACTGGAGCGTGGAGAAGCGCAGCGTCCGAGACGTGCCGCTGGTCAGGCTGCCGGCCGACAGCCCGGTGCCAGAGCGCGGCGACCTCAGCTGCAGGATGCACACGTGTTTCGACGTCTACCGCTGCGGCTTCAACCCCAAGAACAAGATCAAGGTGTACATCTACCCGCTGAAGAAGTACGTGGGCGAGGCGGGTGTCCCGGTGAGCAGCACCATCTCCCGGGAGTACAACGAGCTGCTCACGGCCATCTCAGACAGCGACTACTACACCGACGACGTCACCCGCGCCTGCCTGTTCGTCCCGTCCATCGACCTGCTCAACCAGAACTCGCTCCGCGTGAAGGAGACGGCGCAGGCGCTGGCCCAGCTCTCCAG |
| psiCHECK2-circGLIS3-MUT | ATCTGCATCACCCAGAGCTGGTCCACCAGGCCCAGCCGCTGGCCCTGCCTCAGGCCGCCCTGGAGGAGGATGGGGAGATGGATGACGTTGGGGGCAAGCAAGTAGTACGCTGGATCGACTGCAGTGCCTTGTATGACCAGCAGGAGGAACTTGTGCGGCACATCGAGAAGGTTCATATAGACCAGCGCAAAGGCGAAGACTTCACTT |
| psiCHECK2-  miR-21-3p 2x | AGACAGCCCATCGACTGCTGTTAGACAGCCCATCGACTGCTGTT |
| psiCHECK2-  LEPR-MUT-1 | TTGATTTAGAACTTAAAATAGATGGGTAAATTTGGAATTCAAGACATTGATCTCAGTCCAGTTCAGTTTGGATGTATGATTACTTCTCAGGTCATCTCAAAAGTAGTATTTATGATATCTGGCTTTTGATTTGTCCTTTGTCTGGTCATAAAACATTAAAAAATATGACTGTTGCTCTCAGCACATATCTATTAGTTACCATCAAATTTATAGTAGATAAGTAGTAAACTGCAAATACAATGACTCTAAATATAAAATGTAGCAAAAGTTTTTAGTTTACATTTTATGTCCAAAAATTTCCATTTTATATCCTTTTATAGACACTTCAGCCTATAAATTGAACCATTTCGGTATTCTTTATAATGTCTTGGAGATCTTATATATATTTAACTCTGATCCATATGGGAATTTTCATTTTAGCCTTGAAAATGGCCTAAATGCTAACTTCCATTTCAGTTCAAAG |
| psiCHECK2-LEPR-MUT-2 | TTATGGTTATTATCACCCATATATTTCATTGAGTCCAACATTATGTTAAATGAATTGTCTTTTTATATGGAATATATTTGCCATTATGAATGTTGATTATATCATTATTTCAAGCAATATATCTGAATTCCTTCTATTAGTTTAAGAATTATAAAGTTCAAAATTTTAATTATATGTATAGCATAAAATTGAACACATTTTCTATAGTACTTCATTTAGTTGTGTTAATTTTTCAATAGTAGTAGTCTACAGTTTTGTTTATTTTGCATTTATTTTTTCAATATGTTTACATTGTATGAGTTGAGCTTTTTTGCACATAGATGCTTGCTTGTAGCTGCTTAGCAGGGTTTGTTGTTGTAATGTAACCACTGTTCTTATAAAACTATGATGTACATTTGTTTTGCATCTTTGTCTTCATTCCACATGTCCTATAGTTTATATTTTTATGTGAAGTTATTTATAAGGAATG |
| LEPR | ATGATCAGCCAGAAGTTCTGCGTGGCCCTGCTGCACTGGGAGTTCATCTACGTGATCACCGCCTTCAACCTGGCCTACCCCATCACCCCTTGGAAGTTCAAGCTGAGCTGCATGCCCAGCAACACCACCTACGACTTCCTGTTCCCTCCTGGCATCTCCAAGAACACCTCTAACCTGAATGGCCGCTACGAAGCCGTGGTGGAGACCAAACTGAACTCCTCCGGGACCTACCTGAGCAACCTGAGCAGCAGAACCACCTTCCACTGCTGCTTCTGGAGCGAAGAAGACAAAAACTGCAGCGTGTACACCGATGACATCGAGGGCAAAGCCTTCGTGACCACAGTGAACAGCCTGGTGTTCCAGCAGACCGGCGCCAATTGGAACATCCAGTGCTGGATGAAGGAGGACCTGAAGCTGTTCATCTGCAACATCGAGAGCCTGTTCAAGAACCCATTTAAGAATTACGACCTGAAGGTGCACCTGCTGTACGTGCTGCTGGACGTGCTGGAGGAGAGCCCTCTGCTGCCTCAGAAGGACAGCTTCCAGGTGGTGCAGTGCAACTGCAGCGTGCACGAGTGCTGCGAGTGCCATGTGCCTGTGCCTACCGCCAAACTGAACGATACACTGCTGATGTACCTGAAAATCACCTCCGGCGGCGCCGTGTTCCATTCTCCTCCTATGAGCGCCCAGCCAATCAACGTGGTGAAACCCGACCCTCCTCTGGGGCTGAGAATGGAAATCACCGACACCGGCTCCCTGAAGATCAGCTGGTCCTCCCCTACCCTGGTGCCTTTCCAGCTGCAGTACCAGGTGCAGTATTCTGAAAACAGCACCAAATACATCCGGAAAACAGACGAGATCGTGAGCGCCACCTCCCTGCTGGTGGATTCTGTGCTGCCCGGAAGCTCTTACGGCGCCCAGGTGAGATGCAAGCGGCTGGATGGACTGGGCATCTGGAGCGATTGGAGCGCCCTGCTGACCTTCACCACCCAGGATGTGATTTACTTCCCTCCTAAGATCCTGACCGCCGTGGGGTCTAATATCTCTTTCCACTGCATTTATAAGAACGAGAAGAAGATTGTGAGCTCCAAGAAGATCGTGTGGTGGCTGAACCTGGCCGAGAAAATCCCCCAGAGCCAGTACGACGTGGTGGACGATCACATCAGCAAAGTGACCTTTCCCAACCTGAACGCCACCAAGCCCCGCGGAAAGTTCACCTATGACGCCGTGTACTGCTGTAACGAGCAGGAATGCCACCACCGGTATGCCGAGCTGTACGTGATCGACGTGAACATCAACATCTCCTGCGAAACCGACGGCTACCTGACCAAGATGACCTGCAGGTGGAGCCCCAACGCCATTCAGAGCCTGGCCGGATCTAACCTGCAGCTGAGATATCACCGGTCCAGCCTGTACTGCTCCGACGTGCCTAGCATTCACCCCGTGAGCGAGCCTAAGGACTGCCATCTGCAGAGAGACGGCTTCTACGAGTGTATTTTCCAGCCCATCTTCCTGCTGTCCGGCTACACCATGTGGATCAGGATTAACCACACCCTGGGCTCTCTGGACTCCCCACCTGCCTGTGTGATTCCCGACTCTGTGGTGAAGCCCCTGCCTCCTAGCTCCGTGAAGGCCGAAATTACCGTGAAGATTGGCCTGCTGAAGATCTCCTGGGAAAAGCCCGTGTTCCCCGAAAATAACCTGCAGTTCCAGATCCGGTATGGCCTGTCTGGCAAACAGGTGCAGTGGAAAATGTTCGAAGTGTACGACGCCAAACTGAAGAGCGCCAGCCTGCCAGTGCCCGATCTGTGTGCCGTGTACACAGTGCAGGTGCGGTGCAAGAGCCTGGATGGACTGGGATACTGGAGCAACTGGAGCACCCCTGCCCACACAGTGGTGATGGACGTGAAAGTGCCTATCCGGGGCCCCGAATTTTGGAGACTGATCAGCGAGGACACCACAAAGAAGGAGCGGAACGTGACCCTGCTGTGGAAACCCCTGATGAAAAACGATAGCCTGTGCTCTGTGCGGCGCTACGTGGTGAAGCACCACACAAGCCACAACGGCACCTGGCTGGAAGACGTGGGAAATCACACCAAACTGACCTTCCTGTGGACCGAGCAGGCCCATAGCGTGATGGTGCTGGCCATCAACTCTATTGGAGCCAGCAGCGCCAACTTCAATCTGACCTTTAGCCGGGCCATTAGCAAGGTGAACATCGTGCAGAGCCTGAGCGCCTACCCCCTGAATTCTAGCTGCGTGATTCTGAGCTGGATGCTGTCCCCAAGCGACTACAACCTGATGTACTTTATCCTGGAGTGGAAGATCCTGAACGAGGATAGCGAAATTAAGTGGCTGCGGATTCCTAGCTCCGTGAAGAAGTACTACGTGCACGACTACTTCATCCCCATCGAGAAATACCAGTTCAGCCTGTACCCCATCTTCACCGAGGGCGTGGGAAAGCCTAAGATCATCAATAGCTTCGCCCAGGATGACGAGAAGCACCAGCACGACGCCGACCTGTACGTGATTGTGCCCATCATCATCAGCAGCAGCATCCTGCTGCTGGGGATCTTGTCCGTGAGCCACCAGAGGATGAAGAAGCTGTTTTGGGAGGACGTGCCCAACCCCAAGAACTGCAGCTGGGCCCAGGGACTGAACTTCCAGAAGCCCGAGACCTTCGAGCATCTGTTCATTAAGCACACCGAGAGCACCACCTTCGGCCCTCTGCTGCTGGAACCTGAGACCATCAGCGAGGATATCTCTGTGGACACCTCCTGGAAAAACAAAGACGAGATGGTGCCCGCCACCACCGACGCCCTGCTGCTGACAACACCTGACCTGGAAAAGGGCAGCATCTGTATCTCCGACCAGTGCAGCTCCGCCCAGTTCTCTGAAGCCGAATCCACCGACATCACCTGCGAGGACGAAAGCCGGAGGCAGCCTTCTGTGAAGTATGCCACCCTGCTGAGCAACTCCAAGAGCGGAGAGACCGAAGAGGAGCAGGGCCTGATCAATAGCAGCGTGTCCAAGTGCTTCCTGAGCAACAACAGCCCACCAAAAGACAGCAGCAGCAAACGCAGCTGGGAGATCGAGACCCAGGCCTTCTTCATCCTGAGCGACCAGCACCCCAACATCATCAGCCCCCACCTGCCATTCAGCGAAGGCCTGGATGAGCTGCTGAAGCTGGAGGGAAACTTCCCTGAGGAGAACAACAACGAACGCCCCGTGTACTACCTGGGCGTGACATCTATCAAGAAGCGGGAGAGCGACGTGTTCCTGACAAACGAGAGCCGGGTGCTGAGCCCATTCCCAGCCCATTGTCTGTTCACCGACATTCGGATCTTGCAGGACTCTTGTTCTCACCTGGTGGAGAATAATTTTAACCTGGGCACCTCCGGCCAGAAGACCTTCGTGTCTTACATGCCCCAGTTCCAGACCTGCTCCACCCAGACACAGAAGATCATGGAGAACAAGATGTGCGACCTGACCGTGTGA |

**Table S2.** Oligonucleotide sequences in this study.

| **Fragment name** | **sense, (5′-3′)** | **antisense, (5′-3′)** |
| --- | --- | --- |
| si-NC | UUCUCCGAACGUGUCACGUTT | ACGUGACACGUUCGGAGAATT |
| si-circGLIS3 | CCAACAAGUGUUCGGGGCUTT | AGCCCCGAACACUUGUUGGTT |
| si-circGLIS3# | ACAAGUGUUCGGGGCUCUUTT | AAGAGCCCCGAACACUUGUTT |
| si-LEPR | GGCUAUACAAUGUGGAUUATT | UAAUCCACAUUGUAUAGCCTT |
| mimic-NC | UUCUCCGAACGUGUCACGUTT | ACGUGACACGUUCGGAGAATT |
| miR-21-3p mimic | AACAGCAGUCGAUGGGCUGUCU | ACAGCCCAUCGACUGCUGUUUU |
| inhibitor-NC | CAGUACUUUUGUGUAGUACAA |  |
| miR-21-3p inhibitor | AGACAGCCCAUCGACUGCUGUU |  |

**Table S3.** Primers utilized in this study.

| **Primer name** | **Primer sequences (5′-3′)** | **Size (bp)** |
| --- | --- | --- |
| β-actin | F: ATCGGCAATGAGCGGTTC  R: CGTGTTGGCGTAGAGGTC | 144 |
| circGLIS3  (circRNA32) | F: AGACTTCACTTGTTTCTGGG  R: TGACCACTAGATTGTTGCAG | 181 |
| circRNA1057 | F: AACGCTTCAGTGAACTTGCT  R: ACCGGATTCTTCTGCAACC | 78 |
| circRNA1290 | F: GTCGTCGAAGTCACCTTGTCC  R: CCTGGAATCTTCGCATGTCCC | 156 |
| circRNA1708 | F: AACGCACCAGCATCGG  R: GCCCTCCTGTTTCTACTCC | 140 |
| circRNA2209 | F: AGGCTGCAGGATTTCATAACTGG  R: GGCCCACAGGAAAGTTGTCA | 197 |
| circRNA2627 | F: TGTTTACATAGTCATGGAGC  R: TTTTGTTCTACATACGCAAG | 233 |
| circRNA4491 | F: GCGTCTTCCAGAGAGTCACA  R: CTTCATGTAAAGATAGCACCTT | 130 |
| circRNA5655 | F: CGGCCATCTCAGACAGCGACT  R: ACCCCGGATGTTGTACTTGACC | 196 |
| circGLIS3-convergent | F: CTCGAAGGTACAAGCCATTTA  R: ATTGCCCTCTGTAAGCTAGGA | 255 |
| circGLIS3-divergent | F: AGCAAGCCAGGTCTCTACAGC  R: GCGACCACTCTTGTTTCACGTT | 185 |
| linear GLIS3 | F: ACTCAAGCCGAAGTGGAACA  R: GCAGAAGGTGCAAACCTCTCA | 163 |
| U6 | F: GCTTCGGCAGCACATATACT  R: TTCACGAATTTGCGTGTCAT | 92 |
| PPARγ | F: ATCAAGTTCAAGCACATCAGTC  R: GTCGTTCAAGTCAAGGTTCAC | 156 |
| SREBF1 | F: CTCCGACACCACCAGCATCAAC  R: GCAGCCCATTCATCAGCCAGAC | 122 |
| FASN | F: GGCAAACGGAAAAACGGTGA  R: CTTGGTATTCCGGGTCCGAG | 183 |
| FABP4 | F: AGATGGTGCTGGAATGTGTC  R: ATTCGTTTGGCAGGATCATAGA | 79 |
| C/EBPα | F: AGGAGGACGAAGCGAAGC  R: GTGTGCGATCTGGAACTGC | 134 |
| ACCα | F: CTCCAACCTCAACCACTACGG  R: GGGGAATCACAGAAGCAGCC | 138 |
| LEPR | F: GTGTGCTTCCTGGGTCTTCGTATG  R: GGTAAGAAGGGCACTCCAATCACTC | 92 |
| LEP | F: GAGGGTCACTGGTTTGGACTTCATC  R: TGGAAGGCAGACTGGTGAGGATC | 110 |
| AMPKα1 | F: TTGCGTGTTCGGAGGAAGAATCC  R: GTGGCGTAGCAGTCCCTGATTTG | 148 |
| AMPKα2 | F: ATGATGAGGTGGTGGAGCAGAGG  R: GCCCGTGAGAGAGCCAGAGAG | 137 |
| mmu-circGLIS3  (bta-circGLIS3) | F: TATGACCAGCAGGAGGAACT  R: TGACCACTAGATTGTTGCAG | 246 |
| mmu-PPARγ | F: TGTTCGCCAAGGTGCTCCAG  R: AGGCTCATGTCTGTCTCTGTCTTC | 103 |
| mmu-SREBF1 | F: GGATCGCAGTCTGAGGAGGAG  R: CCAGGAGCCGACAGGAAGG | 147 |
| mmu-FASN | F: TCCTGAAGCCGAACACCTCTG  R: GCGACAATATCCACTCCCTGAATC | 101 |
| mmu-FABP4 | F: TCACCGCAGACGACAGGAAG  R: AAGTCACGCCTTTCATAACACATTC | 150 |
| mmu-C/EBPα | F: CAAGAAGTCGGTGGACAAGAACAG  R: CGTTGCGTTGTTTGGCTTTATCTC | 101 |
| mmu-β-actin | F: ACTGCCGCATCCTCTTCCTC  R: AACCGCTCGTTGCCAATAGTG | 80 |

**Table S4** Primer information of miRNAs utilized in this study.

| **Primer name** | **Primer sequences (5′-3′)** |
| --- | --- |
| miR-21-3p | F: TGCAACAGCAGTCGATGGGCTGTCT |
| miR-151-5p | F: CCTCGAGGAGCTCACAGTCTAGT |
| miR-148a | F: CGCAGCGCTCAGTGCACTACAGAACTT |
| miR-148b | F: TCGAGCGCTCAGTGCATCACAGAACT |
| miR-214 | F: GAACAGCAGGCACAGACAGGCAGT |
| miR-152 | F: GCGCTCAGTGCATGACAGAACTTGGG |

**Table S5.** The sequence information of RNA-FISH.

| **Primer name** | **Primer sequences (5′-3′)** |
| --- | --- |
| circRNA32 probe1 | AGAGCCCCGAACACT+TGTTGGGCTT |
| circRNA32 probe2 | CCAAGAGCCCCGAACACT+TGTTGG |
| circRNA32 probe3 | CCAAAGCCAAGAGCCCCGAACACT+T |

**Table S6.** The information on antibodies.

| **Antibody name** | **Source** | **Dilution ratio** | **Item No.** | **Manufacturers** |
| --- | --- | --- | --- | --- |
| PPARγ | Rabbit | 1:500 | AF7797 | Beyotime |
| SREBF1 | Rabbit | 1:2000 | 14088-1-AP | Proteintech |
| FASN | Rabbit | 1:5000 | ab99359 | Abcam |
| FABP4 | Rabbit | 1:7000 | ab92501 | Abcam |
| C/EBPα | Rabbit | 1:750 | ab40764 | Abcam |
| ACCα | Mouse | 1:500 | ab205883 | Abcam |
| LEPR | Rabbit | 1:1000 | 20966-1-AP | Proteintech |
| LEP | Rabbit | 1:1000 | DF8583 | Affinity |
| AMPKα | Rabbit | 1:500 | AF6423 | Affinity |
| p-AMPKα | Rabbit | 1:500 | D151212 | Sangon Biotech |
| β-actin | Rabbit | 1:5000 | D110001 | Sangon Biotech |
| Goat Anti-Rabbit IgG | Goat | 1:5000 | D110058-0025 | Sangon Biotech |
| Goat Anti-Mouse IgG | Goat | 1:10000 | ab150113 | Abcam |

**Table S7.** Research progress on circRNAs derived from the *GLIS3* gene and their homology comparison with bovine *circGLIS3*

| CircBase-ID | Length | Identitie | Biological Function | Pathway | References |
| --- | --- | --- | --- | --- | --- |
| *mmu_circ_0000943* | 1114 | 80% | Protect against islet β-cell dysfunction and apoptosis in obesity | miR-124-3p/*NeuroD1 or Creb1*  SCOTIN (RNA-binding proteins) | Liu et al.(2023)^1^ |
|  |  |  | Contribute to lipotoxicity-induced beta cell dysfunction, participate in regulating islet EC function | exosomal route | Xiong et al.(2021)^2^ |
|  |  |  | Promote β-cell dysfunction | Encoding Glis3-348aa protein | Xiong et al.(2024)^3^ |
|  |  |  | Ameliorate renal ischemia reperfusion-triggered inflammation and oxidative stress | miR-377-3p/Egr2 | Huang et al.(2022)^4^ |
| *hsa_circ_0006370* | 1114 | 85% | / | / | / |
| *mmu_circ_0007672* | 1322 | 82% | / | / | / |
| *hsa_circ_0007368* | 1322 | 87% | Inhibit the migration and invasion of thyroid cancer cells | miR-146b-3p/ AIF1L | Cao et al.(2023)^5^ |
| *hsa_circ_0002874* | 486 | / | Promote prostate cancer cell proliferation, migration, and invasion | miR-661/MDM2/p53 | Cheng et al.(2024)^6^ |
|  |  |  | Promote intrahepatic cholangiocarcinoma progression | hnRNPA1/G3BP1/NF-κB (RNA-binding proteins) | Kang et al.(2024)^7^ |
|  |  |  | Promotes high-grade glioma invasion | Ezrin phosphorylation | Li et al.(2021)^8^ |
|  |  |  | Promote bladder cancer proliferation | miR-1273f/SKP1/Cyclin D1 | Wu et al.(2021)^9^ |

1. Liu Y, Yang Y, Xu C, Liu J, Chen J, Li G *et al.* Circular RNA circGlis3 protects against islet β-cell dysfunction and apoptosis in obesity. *Nature Communications* 2023;**14**:351.

2. Xiong L, Chen L, Wu L, He W, Chen D, Peng Z *et al.* Lipotoxicity-induced circGlis3 impairs beta cell function and is transmitted by exosomes to promote islet endothelial cell dysfunction. *Diabetologia* 2022;**65**:188–205.

3. Xiong L, Gong Y, Liu H, Huang L, Zeng Z, Zheng X *et al.* circGlis3 promotes β-cell dysfunction by binding to heterogeneous nuclear ribonucleoprotein F and encoding Glis3-348aa protein. *iScience* 2024;**27**:108680.

4. Huang T, Gao Y, Cao Y, Wang Q, Dong Z. Downregulation of mmu_circ_0000943 ameliorates renal ischemia reperfusion-triggered inflammation and oxidative stress via regulating mmu-miR-377-3p/Egr2 axis. *International Immunopharmacology* 2022;**106**:108614.

5. Cao S, Yin Y, Hu H, Hong S, He W, Lv W *et al.* CircGLIS3 inhibits thyroid cancer invasion and metastasis through miR-146b-3p/AIF1L axis. *Cell Oncol* 2023;**46**:1777–1789.

6. Cheng X, Yang H, Chen Y, Zeng Z, Liu Y, Zhou X *et al.* METTL3-mediated m6A modification of circGLIS3 promotes prostate cancer progression and represents a potential target for ARSI therapy. *Cellular & Molecular Biology Letters* 2024;**29**:109.

7. Kang F-P, Chen Z-W, Liao C-Y, Wu Y-D, Li G, Xie C-K *et al.* Escherichia coli‐Induced cGLIS3‐Mediated Stress Granules Activate the NF‐κB Pathway to Promote Intrahepatic Cholangiocarcinoma Progression. *Advanced Science* 2024;**11**:2306174.

8. Li Y, Chen J, Chen Z, Xu X, Weng J, Zhang Y *et al.* CircGLIS3 Promotes High-Grade Glioma Invasion via Modulating Ezrin Phosphorylation. *Frontiers in Cell and Developmental Biology* 2021;**9**:663207.

9. Wu S, Yang J, Xu H, Wang X, Zhang R, Lu W *et al.* Circular RNA circGLIS3 promotes bladder cancer proliferation via the miR-1273f/SKP1/Cyclin D1 axis. *Cell Biology and Toxicology* 2021;**38**:129.
