# Supplementary material for: CircGLIS3 Inhibits Intramuscular Adipogenesis and Alleviates Skeletal Muscle Fat Infiltration
Source: J Cachexia Sarcopenia Muscle. 2025 Jul 30;16(4):e70009. doi: 10.1002/jcsm.70009 (PMC12308225; doi:10.1002/jcsm.70009)
Supplement: Supplementary file 2 — Material S1. The details of the supplementary materials and methods are provided. [file JCSM-16-e70009-s003.docx]

**Supporting Information**

**WGCNA analysis**

Our laboratory has previously conducted RNA-seq on circRNAs at four stages of adipogenic differentiation (0, 3, 6, and 9 d) from intramuscular preadipocytes of Qinchuan beef cattle. The data obtained from this study have been deposited in the NCBI GEO database under the accession number GSE185850. A total of 14,070 circRNAs were identified through bioinformatic analyses for further investigation ^1^. StringTie was used to calculate the fragments per kilobase of transcript per million mapped reads (FPKM). Then, log2 (FPKM+1) was used to standardise the expression matrix, and circRNAs with the standard deviation (SD) of expression levels greater than 0.75 in all samples were selected for downstream analysis. The “WGCNA V1.69” package in RStudio 4.0 was used to construct the weighted co-expression network ^2^. Following the principle of a scale-free network, the optimal soft threshold (β) in the co-expression matrix was selected, and the co-expression matrix was transformed into an adjacency matrix. A topological overlap matrix (TOM) was generated based on the adjacency matrix. Genes exhibiting similar expression patterns were classified into gene modules through average linkage hierarchical clustering (minModuleSize = 20) using the topological difference matrix (disTOM= 1-TOM), and the modules with more than 50% similarity were merged. The hierarchical clustering method was employed to create a dendrogram and calculate between module characteristic genes and different differentiation times of adipocytes. Modules with high correlation coefficients and small significant P-values were identified as key areas of interest. Additionally, a heatmap was employed to visualise the expression of cirRNAs across samples.

**Cell isolation, culture, and differentiation**

Primary bovine intramuscular preadipocytes were isolated from the longissimus dorsi muscle of three newborn cattle and subsequently inoculated into cell culture dishes, as previously described ^3^. Primary bovine intramuscular preadipocytes were cultured in a complete medium consisting of 84% DMEM-F12 (Hyclone, USA), 15% fetal bovine serum (PAN-Biotech, Germany), and 1% penicillin/streptomycin (Hyclone, USA)) at 37℃ and 5% CO_2_. The medium was changed every 2 days. When the intramuscular preadipocytes reached 100% confluence, the medium was changed to the adipogenic differentiation induction medium (complete medium containing 10 μg/mL insulin (Sigma), 1 μM dexamethasone (Sigma), 0.5 mM 3-isobutyl-1-methylxanthine (Sigma), and 2 μM rosiglitazone (Sigma)) for 2 days. Subsequently, the medium was changed to a complete medium containing 10 μg/mL insulin, with further medium changes every 2 days.

HEK293T (established mouse preadipocyte cell line) and 3T3-L1 (established human embryonic kidney 293 cell line) cells were cultured in a complete medium consisting of 89% DMEM-high glucose (Hyclone, USA), 10% fetal bovine serum (PAN-Biotech, Germany), and 1% penicillin/streptomycin (Hyclone, USA) at 37℃ and 5% CO_2_. The medium was changed every 2 days. Adipogenic differentiation of 3T3-L1 cells was conducted based on the adipogenic differentiation protocol of primary bovine intramuscular preadipocytes.

HEK293T (ATCC CRL-1126) and 3T3-L1 (ATCC CL-173) were purchased from the American Type Culture Collection (ATCC).

**Vector construction, siRNAs, and cell transfection**

The full-length oligonucleotide sequence of circGLIS3 was synthesized and cloned into the pcD25-ciR and pcD5-ciR vectors (overexpression vectors) (Geneseed, Guangzhou, China). We used the same method to construct pcDNA3.1-LEPR (pcD3.1-LEPR) (Tsingke, Beijing, China). We then constructed psiCHECK2-circGLIS3-WT (pCK-circGLIS3-WT) with the full-length circGLIS3 into the psiCHECK2 vector (Promega, Madison, WI, USA). We constructed psiCHECK2-LEPR-WT-1 (pCK-LEPR-WT-1) and psiCHECK2-LEPR-WT-2 (pCK-LEPR-WT-2) by cloning a segment of the LEPR 3'UTR containing the miR-21-3p binding site. We used the same method to construct psiCHECK2-circGLIS3-MUT (pCK-circGLIS3-MUT), psiCHECK2-LEPR-MUT-1 (pCK-LEPR-MUT-1), and psiCHECK2-LEPR-MUT-2 (pCK-LEPR-MUT-2) with mutation sites paired to miR-21-3p. Furthermore, the miR-21-3p biosensor (miR-21-3p sensor) was constituted by inserting the reverse complement repeats of the miR-21-3p seed region into the psiCHECK-2 vector. The small interfering RNA (siRNA) was synthesized by GenePharma Biol (Suzhou, China), and the miR-21-3p mimics and inhibitors were synthesized by Ribo Biol (Guangzhou, China). The sequence information is detailed in Table S1 and S2.

According to the manufacturer’s instructions, Lipofectamine 3000 reagent (Invitrogen, Carlsbad, CA, USA) was used for all transient transfections. Primary bovine intramuscular preadipocytes and 3T3-L1 cells were transfected at 90% confluence for experiments related to cell differentiation. HEK293T cells were transfected at 50% confluence for the luciferase reporter assay.

***In vivo* animal studies**

The C57BL/6 mice were purchased from SPF (Beijing) Biotechnology Co., Ltd. Sixty-three male mice (8 weeks old, initial body weight approximately 20 g) were randomly divided into two groups: one group of 18 mice was fed a basal diet, and one group of 45 mice was fed a high-fat diet (HFD). The HFD group was fed with 60% kcal from fat, 20% kcal from carbohydrate, and 20% kcal from protein (XTHF60-1, Xietong Pharmaceutical Bio-Engineering Co.,Ltd., Jiangsu, China). After three months of feeding (the body weight of mice in the HFD group reached approximately 30 g), three mice from each group were randomly selected to harvest the vastus lateralis (VL) muscle samples, which were snap-frozen in 2‐methylbutane for 20 s and stored at -80°C until histology analysis ^4^.

After successfully establishing the mouse model of intramuscular fat deposition, we randomly divided the remaining 42 mice in the HFD group into two groups: one group of 15 mice was injected into the left hind limb muscle (VL and GAS) with pcD5-NC (pcD5-NC group), and one group of 27 mice (twelve of these mice were selected to verify the overexpression efficiency of *circGLIS3* after injection of pcD5-circGLIS3) was injected into the left hind limb muscle (VL and GAS) with pcD5-circGLIS3 (pcD5-circGLIS3 group). The 15 mice remaining on the basal diet were injected into the left hind limb muscle (VL and GAS) with 5% glucose (control group). All the mice were injected six times, with one injection every 5 days. At each injection time point, each mouse was weighed individually, and both the provided feed and the remaining feed were weighed to determine daily feed intake. After the injection experiment, the left hind limb muscles (VL and GAS) were harvested. The left hind limb muscles (VL and GAS) of six mice were randomly selected, snap-frozen in 2‐methylbutane for 20 s at the optimal length, and stored at -80°C for histology analysis. The remaining muscle tissue samples were snap-frozen in liquid nitrogen and subsequently stored at -80°C until RNA and protein extraction.

The configuration system of the plasmid mixture was as follows: 6.25 μg of plasmid was diluted in 12.5 μL of 5% glucose solution, and 12.5 μL of Entranster-in vivo DNA transfection reagent (Engreen Biosystem Co., Ltd.) was diluted in 12.5 μL of 5% glucose solution. Subsequently, the two solutions were combined.

**H&E staining and immunofluorescence staining**

VL muscles from mice were fixed in 4% formalin for 24 h at room temperature. The tissues were then embedded in paraffin and sectioned into 4‐μm slices. For haematoxylin and eosin (H&E) staining, the sections were deparaffinized and rehydrated, and the nuclei were stained with haematoxylin for 15 min. After rinsing in running tap water, the sections were stained with eosin for 1 min, dehydrated, mounted, and captured. Whole-slide digital images were obtained using the the Olympus VS200. Scanned images of H&E staining were analysed by Image-Pro Plus 6.0 to quantify the proportion of adipocyte.

**Immunohistochemistry and immunostaining**

Frozen sections were fixed in acetone for 10 min on ice and cells were fixed on coverslips with 4% paraformaldehyde for 15 min, washed with PBS, and treated with 0.3% Triton X-100/PBS at room temperature for a further 20 min. Sections or cells were subsequently incubated with 5% (vol/vol) goat serum/PBS and 10% BSA at room temperature for 1 h, followed by incubation with primary antibodies (diluted in 5% goat serum/PBS/0.1% Triton X-100) for 2 h. Sections or cells were then washed in PBS and incubated with secondary antibodies for 1 h. Primary and secondary antibodies were as follows: anti-Perilipin-1 (1:200, Proteintech) and Alexa Fluor 488 goat anti-rabbit IgG (1:400, Proteintech).

**RNA sequencing (RNA-seq) analysis**

The intramuscular preadipocytes on the third day of differentiation, following interference with circGLIS3 or overexpression of miR-21-3p, were collected, and total RNA was extracted from each sample using RNAiso Plus (Takara). Subsequently, the RNA samples were sent to Hangzhou Biotechnology Technologies Co., Ltd. (Lc-Bio, China) for RNA-seq. Paired-end RNA-seq was performed using the Illumina NovaSeq^TM^ 6000 platform with PE150 mode. The detailed RNA-seq procedures were outlined in previous studies ^1^. A fold change (FC) ≥ 2 (absolute value of log2FC ≥ 1) and a false discovery rate (FDR) < 0.05 (adjusted *P*-value) were set as the threshold criteria for screening differentially expressed genes (DEGs). Kyoto Encyclopedia of Genes and Genomes (KEGG enrichment analysis was performed using OmicStudio tool.

**Nuclear and cytoplasmic RNA separation and the RNA fluorescence in situ hybridization (RNA-FISH) assay**

Nuclear RNA and cytoplasmic RNA were isolated from cells using the Nucleoprotein Extraction Kit (Invitrogen, Waltham, MA, USA). These RNAs were then reverse transcribed into cDNA to further assess the expression levels of *circGLIS3* and linear *GLIS3* mRNA using qRT-PCR or agarose gel electrophoresis. According to the manufacturer's instructions and previous research, the RNA-FISH assay was performed using the [Fluorescent In Situ Hybridization Kit](https://www.ribobio.com/product_detail/?sku=C10910) (Ribo Bio). A mixture of three probes (GenePharma) was used for RNA-FISH. The probes were designed to target the junction site of *circGLIS3* and specifically bind to *circGLIS3* (Table S5).

**Dual-luciferase reporter assay**

When the confluence of HEK293T cells seeded in a 96-well plate reached approximately 50%, the luciferase reporter plasmid, the miR-21-3p mimic, or pcD5-circGLIS3, was co-transfected. Following the manufacturer's instructions, [Renilla](https://www.sciencedirect.com/topics/biochemistry-genetics-and-molecular-biology/renilla) and firefly luciferase activities were measured using the Dual-Luciferase Reporter Assay System (Promega, Madison, WI, USA) at 48 h post-transfection.

**RNA immunoprecipitation (RIP) assay**

According to the manufacturer's instructions, the RIP assay was performed using the Magna RIP™ RNA-Binding Protein Immunoprecipitation Kit (Millipore). Briefly, intramuscular preadipocytes (diff-3 d, 1×10^7^) were collected, lysed in RIP lysis buffer, and incubated on ice for 5 min. The lysed cell suspension was then incubated with 50 Dynabeads Protein A/G overnight at 4°C and then divided into two equal parts. The lysed cell suspension with Dynabeads was incubated with 5 μg IgG antibody (A7016, Beyotime) or AgO2 (BM4920, Boster Biological Technology, Pleasanton, USA) with rotation for 30 min at room temperature. The RNA-protein complex was then incubated with 150 μL of proteinase K buffer for 30 min at 55°C to remove DNA and proteins, and RNA was isolated. Finally, reverse transcription of mRNAs and miRNAs was performed, and qRT-PCR determined the abundance of *circGLIS3* and miR-21-3p.

1. Ma X, Yang X, Zhang D, Zhang W, Wang X, Xie K *et al.* RNA-seq analysis reveals the critical role of the novel lncRNA BIANCR in intramuscular adipogenesis through the ERK1/2 signaling pathway. *J Anim Sci Biotechnol* 2023;**14**:21.

2. Wang J, Li B, Yang X, Liang C, Raza SHA, Pan Y *et al.* Integration of RNA-seq and ATAC-seq identifies muscle-regulated hub genes in cattle. *Front Vet Sci* 2022;**9**:925590.

3. Li P, Wang Y, Zhang L, Ning Y, Zan L. The Expression Pattern of PLIN2 in Differentiated Adipocytes from Qinchuan Cattle Analysis of Its Protein Structure and Interaction with CGI-58. *International Journal of Molecular Sciences* 2018;**19**:1336.

4. Liu K, Yu W, Wei W, Zhang X, Tian Y, Sherif M *et al.* Melatonin reduces intramuscular fat deposition by promoting lipolysis and increasing mitochondrial function. *J Lipid Res* 2019;**60**:767–782.
