# Supplementary figures and images for: CircGLIS3 Inhibits Intramuscular Adipogenesis and Alleviates Skeletal Muscle Fat Infiltration
Source: J Cachexia Sarcopenia Muscle. 2025 Jul 30;16(4):e70009. doi: 10.1002/jcsm.70009 (PMC12308225; doi:10.1002/jcsm.70009)

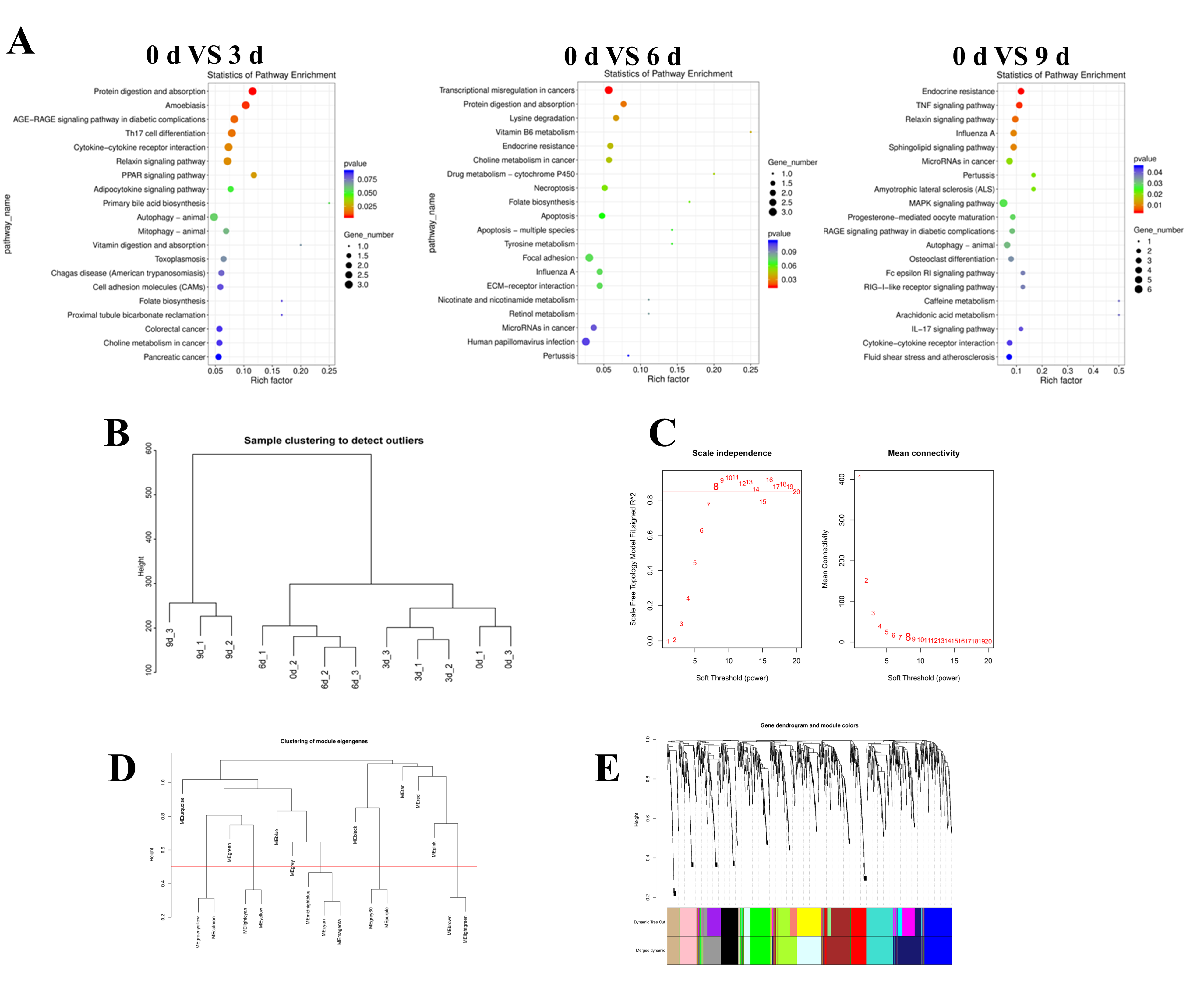

Supplement: Supplementary file 12 — Figure S1. (A) KEGG pathway analysis. (B) Hierarchical clustering tree of all samples. (C) Determination of soft‐thresholding power. When the optimal soft‐threshold was chosen 8, with R 2 = 0.85 and mean connectivity < 100, the network was scale‐free topology. (D) Hierarchical cluster analysis of different modules. The red line represents a cut height of 0.5 to merge modules with more than 50% similarity. (E) Hierarchical clustering of genes with dissimilarity based on topological overlap is shown in the detected and merged modules. [file JCSM-16-e70009-s006.tif]

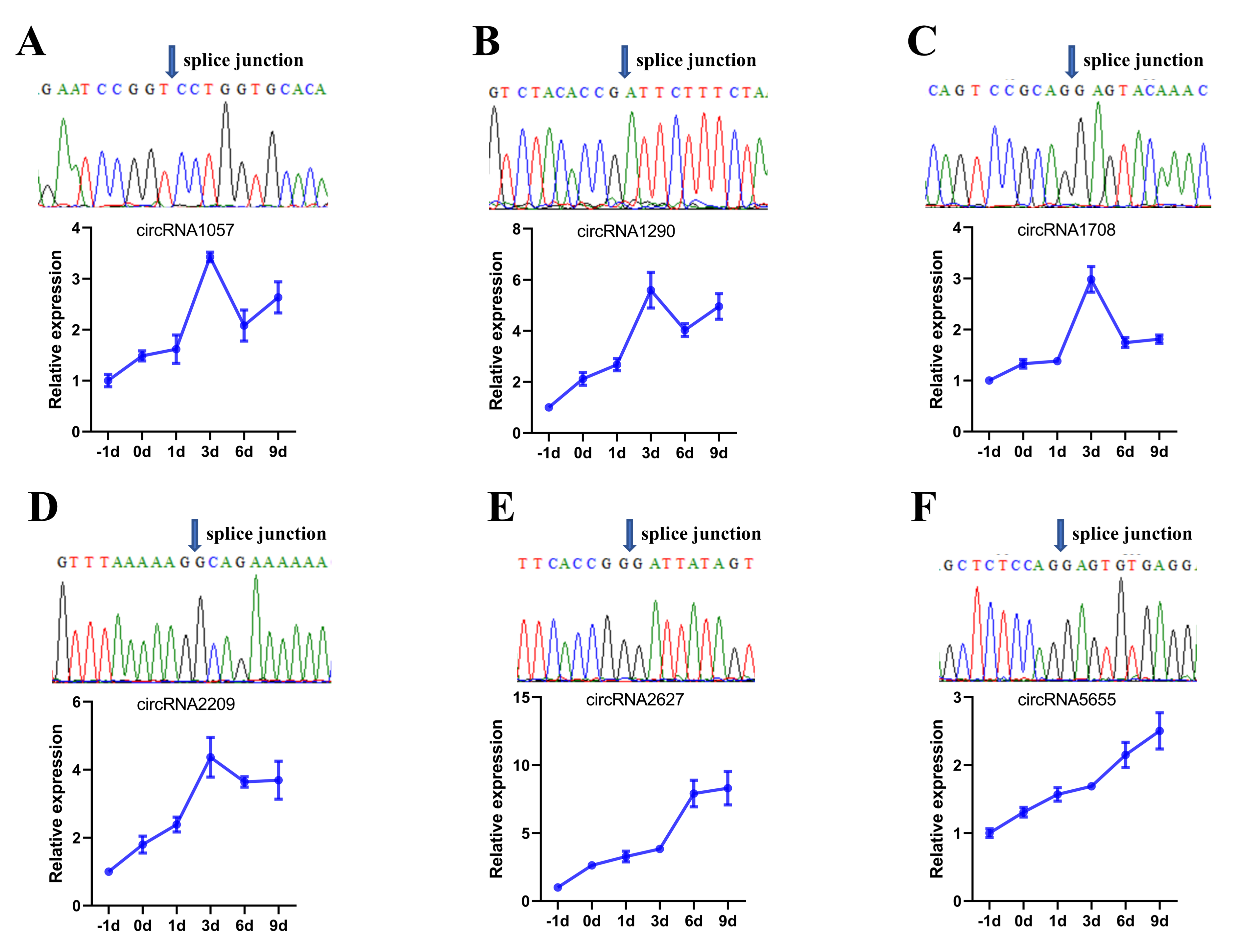

Supplement: Supplementary file 13 — Figure S2. (A–F) Sanger sequencing of candidate circRNAs confirmed the back‐splicing junction sequence (above), and qRT‐PCR (n = 6) detected the expression of candidate circRNAs in different developmental stages of primary bovine intramuscular preadipocytes (below). [file JCSM-16-e70009-s012.tif]

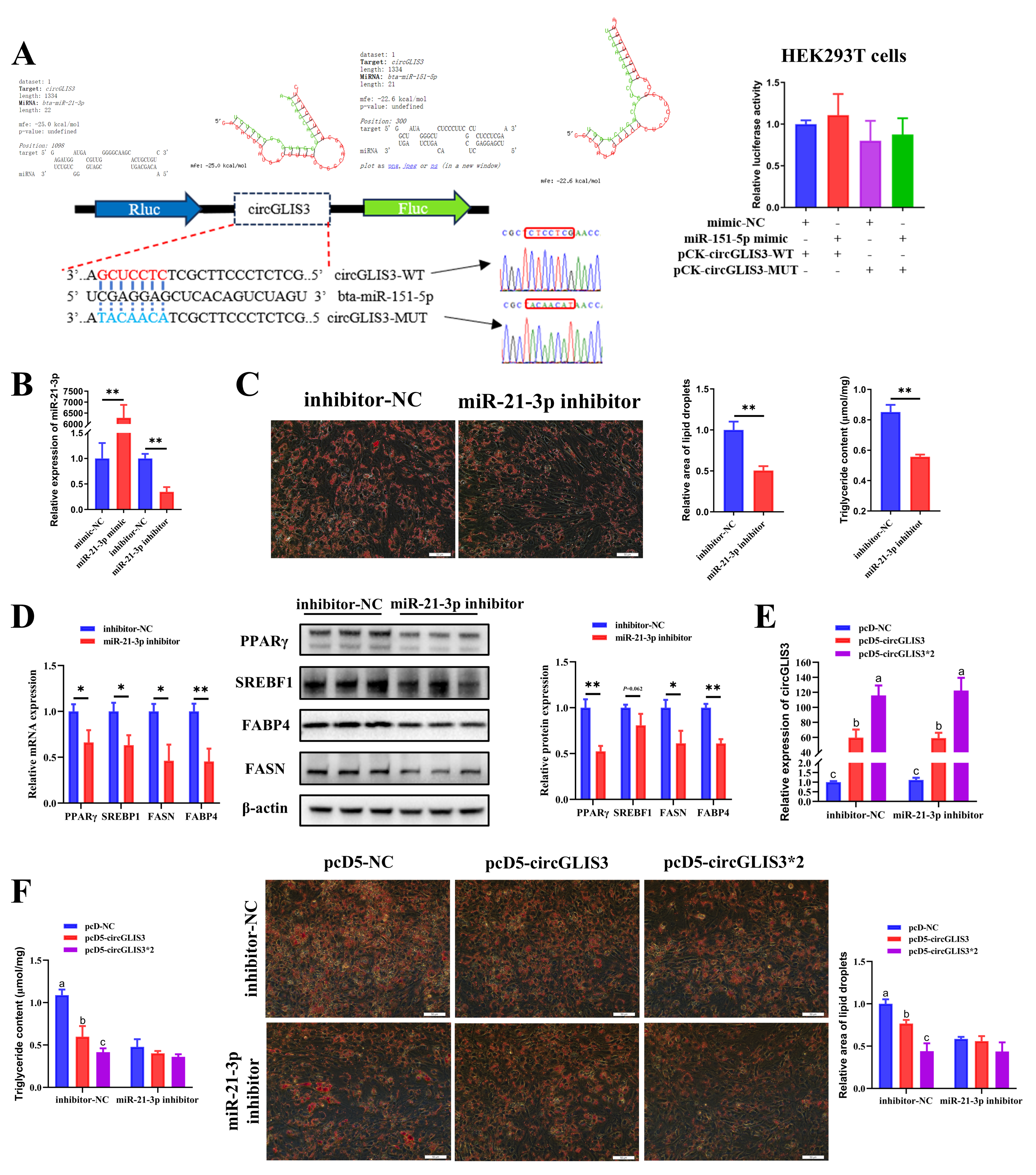

Supplement: Supplementary file 14 — Figure S3. (A) Bioinformatics analysis was used to predict the binding between circGLIS3 and miR‐21‐3p or miR‐151‐5p. The construction of the luciferase reporter vector and related experiments were performed using HEK293T cells (n = 3). (B) After transfecting miR‐21‐3p mimic or inhibitor into primary bovine intramuscular preadipocytes, the expression efficiency of miR‐21‐3p interference and overexpression was determined by qRT‐PCR (n = 6) on the third day of differentiation. (C) Lipid droplet content (determined by Oil Red O staining, n = 9, scale bar 50 μm) and triglyceride content (n = 3) were determined on the sixth day of differentiation in primary bovine intramuscular preadipocytes. (D) Relative mRNA and protein expression levels of PPARγ, SREBF1, FASN and FABP4 were analysed by qRT‐PCR (n = 6) and Western blot (n = 3) on the third day of differentiation in primary bovine intramuscular preadipocytes. (E) Under miR‐21‐3p knockdown conditions, primary bovine intramuscular preadipocytes were transfected with varying doses of pcD5‐circGLIS3, the expression of circGLIS3 was determined by qRT‐PCR (n = 6) on the third day of differentiation. (F) Under miR‐21‐3p knockdown conditions, primary bovine intramuscular preadipocytes were transfected with varying doses of pcD5‐circGLIS3, triglyceride content (n = 3) and the lipid droplet content (determined by Oil Red O staining, n = 9, scale bar 50 μm) were determined on the sixth day of differentiation. Results are presented as the means ± SD, *p < 0.05; **p < 0.01; different lowercase letters indicate significant differences (p < 0.05). [file JCSM-16-e70009-s013.tif]

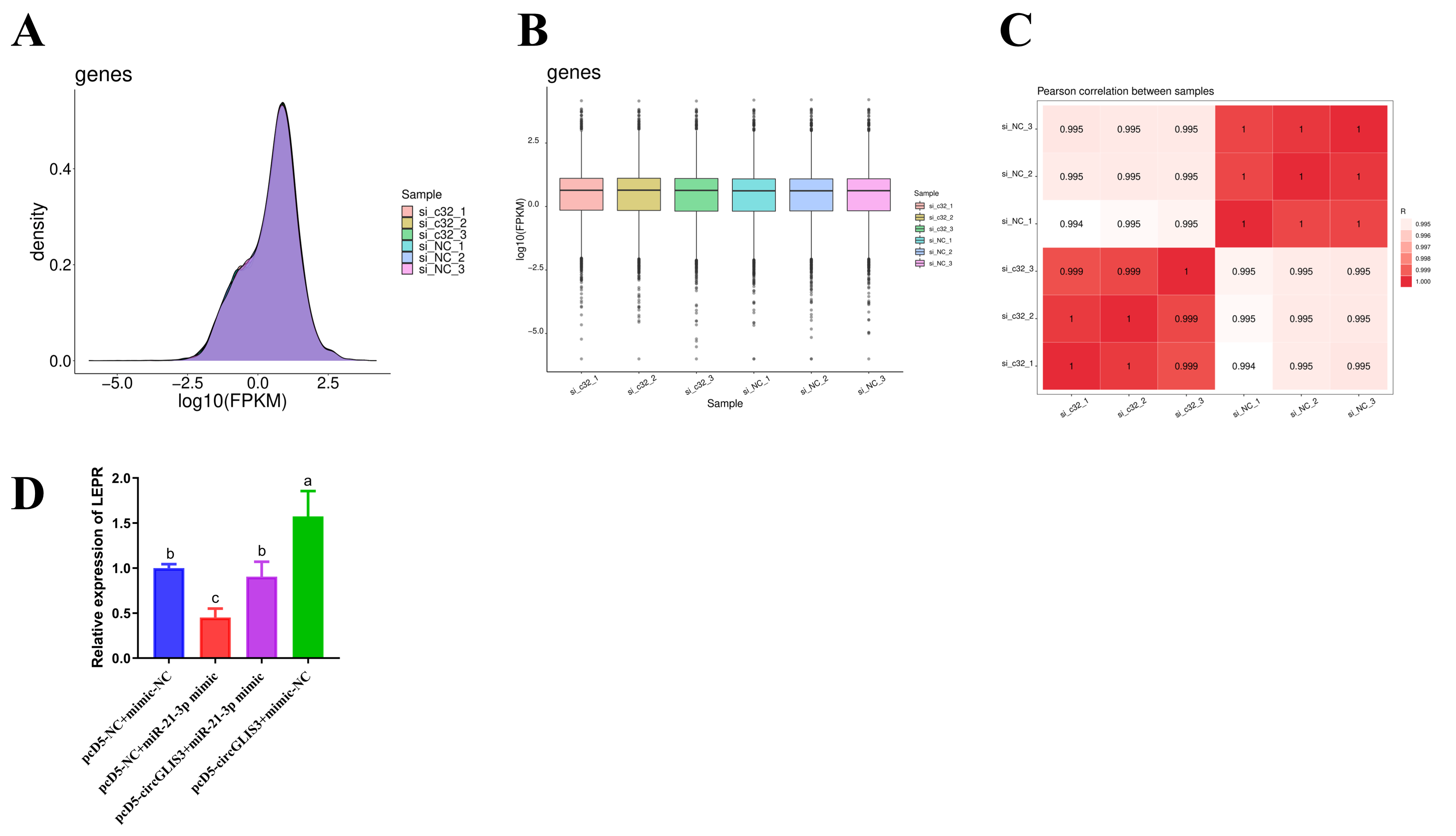

Supplement: Supplementary file 15 — Figure S4. Transcriptome analysis was performed on the third day of differentiation after transfection with si‐circGLIS3 in primary bovine intramuscular preadipocytes. (A–C) Correlation analysis between samples. (D) The pcD5‐circGLIS3 was co‐transfected with the miR‐21‐3p mimic into intramuscular preadipocytes, and relative mRNA expression levels of LEPR were analysed on the third day after differentiation (n = 6) (below). Results are presented as the means ± SD, different lowercase letters indicate significant differences (p < 0.05). [file JCSM-16-e70009-s001.tif]

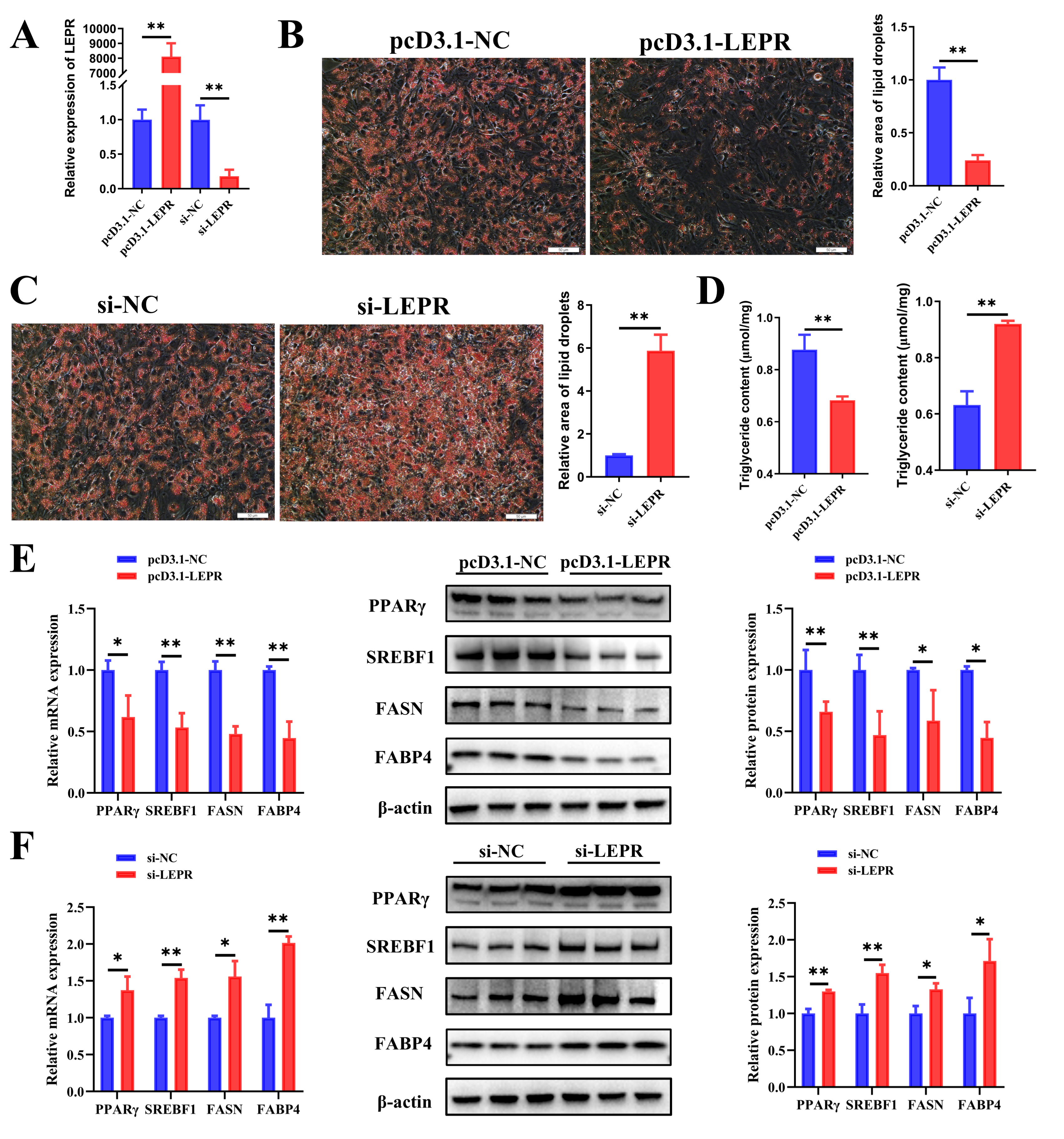

Supplement: Supplementary file 16 — Figure S5. LEPR inhibits adipogenesis of primary bovine intramuscular preadipocytes, and circGLIS3 overexpression reverses the adipogenesis promotion induced by si‐LEPR. (A) qRT‐PCR (n = 3) detected the expression of LEPR on the third day of differentiation after transfection with pcD3.1‐LEPR or si‐LEPR. (B,C) Oil Red O staining (n = 9, scale bar 50 μm) evaluated the lipid droplet content of intramuscular adipocytes on the sixth day of differentiation. (D) Determination of triglyceride content (n = 3) on the sixth day of differentiation after transfection with pcD3.1‐LEPR or si‐LEPR. (E,F) Relative mRNA and protein expression levels of PPARγ, SREBF1, FASN, FABP4 and C/EBPα were analysed by qRT‐PCR (n = 6) and Western blot (n = 3) on the third day of differentiation. Results are presented as the means ± SD, *p < 0.05; **p < 0.01. [file JCSM-16-e70009-s010.tif]
